# Characterization of Spirulina‐derived extracellular vesicles and their potential as a vaccine adjuvant

**DOI:** 10.1002/jex2.70025

**Published:** 2024-12-12

**Authors:** Mohammad Farouq Sharifpour, Suchandan Sikder, Yide Wong, Na'ama Koifman, Tamara Thomas, Robert Courtney, Jamie Seymour, Alex Loukas

**Affiliations:** ^1^ Australian Institute of Tropical Health and Medicine James Cook University Smithfield Queensland Australia; ^2^ Centre for Microscopy and Microanalysis The University of Queensland St Lucia Queensland Australia

**Keywords:** adjuvant, extracellular vesicle, immune response, spirulina, vaccine

## Abstract

Spirulina is an edible cyanobacterium that increasingly gaining recognition for it untapped potential in the biomanufacturing of pharmaceuticals. Despite the rapidly accumulating information on extracellular vesicles (EVs) from most other bacteria, nothing is known about Spirulina extracellular vesicles (SPEVs). This study reports the successful isolation, characterization and visualization of SPEVs for the first time and it further investigates the potential therapeutic benefits of SPEVs using a mouse model. SPEVs were isolated using ultracentrifugation and size‐exclusion‐chromatography. Cryo‐Transmission Electron Microscopy revealed pleomorphic outer‐membrane‐vesicles and outer‐inner‐membrane‐vesicles displaying diverse shapes, sizes and corona densities. To assess short‐ and long‐term immune responses, mice were injected intraperitoneally with SPEVs, which demonstrated a significant increase in neutrophils and M1 macrophages at the injection site, indicating a pro‐inflammatory effect induced by SPEVs without clinical signs of toxicity or hypersensitivity. Furthermore, SPEVs demonstrated potent adjuvanticity by enhancing antigen‐specific IgG responses in mice by over 100‐fold compared to an unadjuvanted model vaccine antigen. Mass‐spectrometry identified 54 proteins within SPEVs, including three protein superfamily members linked to the observed pro‐inflammatory effects. Our findings highlight the potential of SPEVs as a new class of vaccine adjuvant and warrant additional studies to further characterize the nature of the immune response.

## 1 INTRODUCTION

Spirulina is a widely accepted name for several species of blue‐green algae from the genera *Arthrospira* and *Limnospira* within the Cyanobacteria phylum (Nowicka‐Krawczyk et al., 2019). Spirulina is known for its high nutritional values due to its diverse chemical composition and has attracted the industry label of ‘superfood’. Being a source of a diverse range of organic bioactive compounds such as vitamins, peptides and minerals, Spirulina has also been recognized for its therapeutic benefits (Lafarga et al., 2020; Ragaza et al., 2020). Due to these characteristics, as well as the safety and relative ease of biomass scalability, Spirulina is now the most extensively produced microalgae in large‐scale production (Sili et al., 2012; Wu et al., 2016).

Many studies attributed various therapeutic effects to Spirulina in the last few decades. Blood lipid reduction, weight loss and diabetes control, anti‐inflammatory effects, the abundance of antioxidants and anti‐cancer effects, controlling hypertension and positive cardiovascular effects are amongst the attributed medicinal properties of this microalgae (Dinicolantonio et al., 2020; Hu et al., 2019; Martínez‐Sámano et al., 2018; Silva et al., 2021; Wu et al., 2016). Owing to its immunomodulatory properties, Spirulina has been effectively used as an oral therapeutic against metabolic syndromes such as hypertension, atherosclerosis, several types of cancer, infectious diseases such as influenza, allergic rhinitis and neurodegenerative diseases, for example, Alzheimer's disease (Chen et al., 2016; Kulshreshtha et al., 2008; Nawrocka et al., 2017; Selmi et al., 2011).

Extracellular vesicle (EV) is a general term given to a diverse class of nanoscale vesicles enclosed with bilayer phospholipid membranes that are released by organisms across all life kingdoms. EVs are variable in size, shape, biogenesis, surface molecules and cargo and serve as intra‐ or inter‐species cell‐cell communication mediators for many biological interactions (Schorey et al., 2015; Van Niel et al., 2018). In the last few years, EVs have been reported from a number of prokaryotic and eukaryotic microalgae species (Adamo et al., 2021; Hu et al., 2024; Picciotto et al., 2021). While research in this field is in its infancy, it has been suggested that there are similarities between EVs isolated from cyanobacterial microalgae and from Gram‐negative bacteria, including aspects of their composition, formation processes and biological roles. Gram‐negative bacteria produce two main types of EVs: Outer membrane vesicles (OMVs) and outer‐inner membrane vesicles (OIMVs) (Lima et al., 2020). While the morphology and general biology of Cyanobacteria EVs have been relatively well studied (Usui et al., 2022), only a limited number of comprehensive proteomic studies have been performed on EVs produced by Cyanobacteria despite the diversity of the phylum. Previous studies have mainly focused on the secretome or exoproteome in general or on the membrane vesicles from other phyla (Fadeev et al., 2022).

Although a considerable source of natural products with therapeutic potential, no study has yet investigated the therapeutic effects of EVs from cyanobacteria, except for one report on wound topical healing bioactivity of *Synechococcus elongatus* EVs through the promotion of interleukin‐6 expression (Yin et al., 2019).

In this study, we present the first report on the isolation of EVs from Spirulina using an easily reproducible method and the subsequent molecular characterization of the isolated EVs using various experimental techniques. We examined the early and late immune cell responses to Spirulina EVs (SPEVs) after injecting them into mice and further investigated the adjuvanticity properties of SPEVs by immunizing mice with a *Schistosoma haematobium* vaccine antigen.

## 2 MATERIAL AND METHODS

### 2.1 *Spirulina* culture and maintenance

Xenic *Spirulina* microalgae stock was obtained from a commercial source (*Spirulina* Grow Co.—Australia). The stock was maintained and expanded in Zarrouk's medium (Morist et al., 2001) in 500 mL transparent Erlenmeyer flasks inside a home‐made photobioreactor at room temperature with a 150 μ°E constant, blue‐shifted white light irradiated from adjustable LED lights (Aqua Illumination Hydra 64 HD) and air injection for agitation.

### 2.2 Production of axenic culture

**FIGURE 1:**
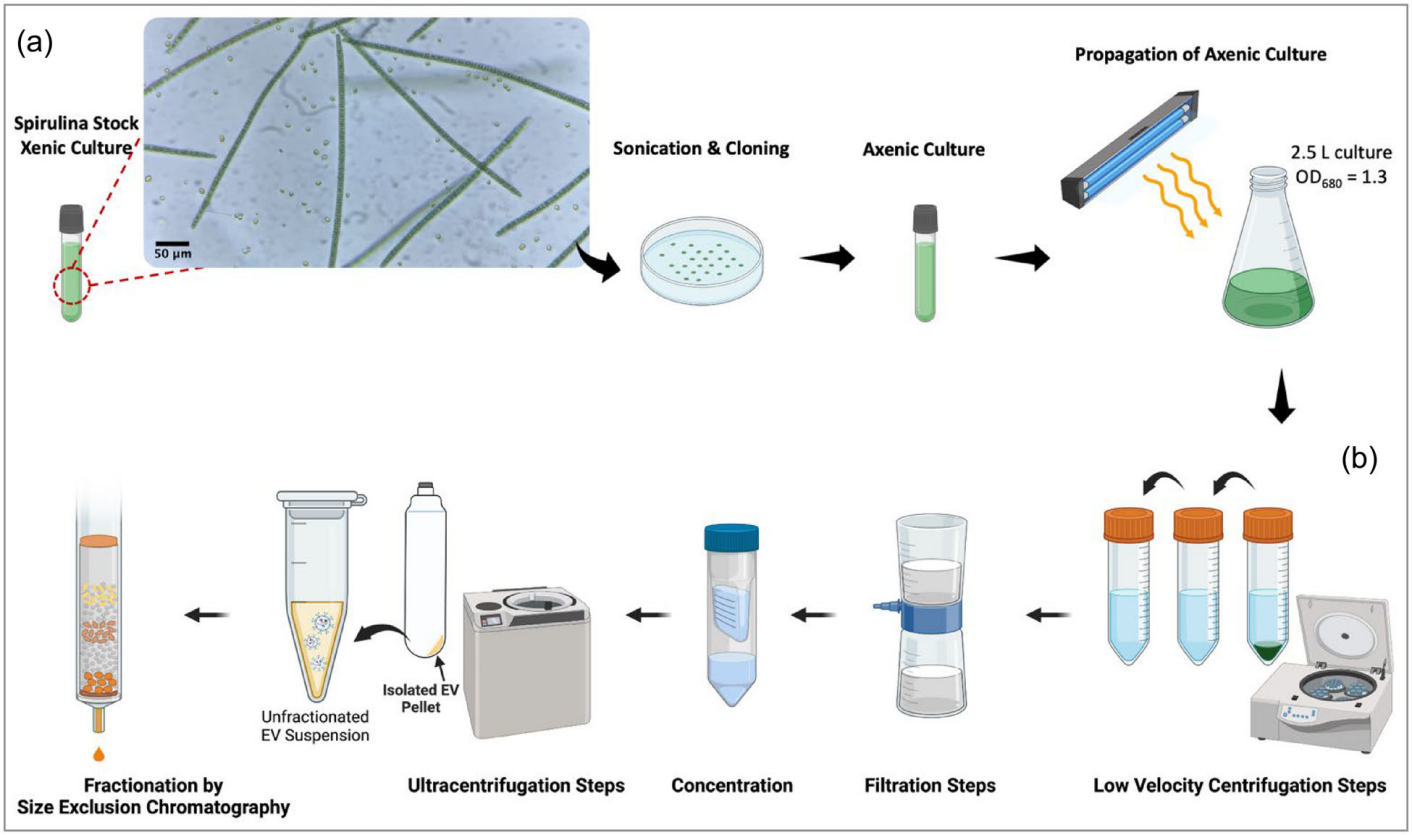
Culture and axenisation of *Spirulina* organism, and extracellular vesicle isolation. (a) The original xenic culture of the *Spirulina* stock was axenised by sonication and cultivating on agar medium in the presence of kanamycin. The axenic culture was then propagated in Zarrouk's medium in photobioreactors. (b) When *Spirulina* reached the lag phase in growth, the cell suspension went through a series of low‐velocity centrifugation steps; the supernatant was collected and passed through several filters and concentrated with 100 kDa cut‐off filters. Ultracentrifugation precipitated EVs in a pellet. The EV pellet was resuspended in water (or PBS) and ultracentrifuged several times in order to remove any soluble proteins. Fractionation was then performed by size exclusion chromatography. EV, extracellular vesicle.

*Spirulina* cultures are naturally accompanied by other symbiotic microorganisms. We aimed to produce an axenic culture in order to isolate pure SPEVs. Xenic *Spirulina* cultures were grown in Zarrouk's medium in the presence of 100 µg/mL kanamycin to reach OD_680_ = 0.1. *Spirulina* filaments were pelleted by centrifugation (1000 × *g*, 10 min, RT) and resuspended in 10 mL sterile MilliQ water. The concentrated *Spirulina* suspension was sonicated for 5 min. 100–200 µL of the sonicated threads were spread out on Zarrouk's agar medium in Petri dishes (Zarrouk's medium + 15% Bacto agar) and sealed with parafilm to prevent the agar medium from drying out. The Petri dishes were incubated at room temperature under consistent light for 4 weeks. Cast‐out single colonies were selected on the plate, transferred to Zarrouk's medium with 100 µg/mL kanamycin in a 6‐well plate and incubated at room temperature under consistent light. After 3 weeks, when the OD_680_ reached 0.3, the axenic *Spirulina* (∼3 mL) were transferred to a 5 L Zarrouk's medium in the presence of kanamycin and incubated, as mentioned before (Figure 1a).

### 2.3 Phylogenetic analysis

To track the genus and species of the *Spirulina* sample (acquired from *Spirulina* Grow Co), the genomic DNA was extracted from *Spirulina* filaments using PureLink Genomic DNA Mini Kit (Invitrogen). A pair of primers was designed for the 16s‐rRNA sequence (MFS‐P1 > AAGGAGGTGATCCAGCCACAC; and MFS‐P2 > GCTTAACACATGCAAGTCGAACGG) using SnapGene software (from Dotmatics; available at snapgene.com) with a predicted PCR product size of 1443 bp. The PCR was performed using Q5 High‐Fidelity DNA Polymerase (NEB), and the expected band was observed. The band was excised from the gel, purified using the QIAquick Gel Extraction Kit and submitted to the Australian Genome Research Facility (AGRF, Brisbane Australia) for Sanger sequencing. The forward and reverse sequencing data was combined using SnapGene Software (www.snapgene.com). Phylogenetic analysis was performed using the BLAST (Basic Local Alignment Search Tool) on the NCBI website. Pairwise distance of the highly similar species was performed using MEGA 11 (Molecular Evolutionary Genetics Analysis) software (Stecher et al., 2020).

### 2.4 EV isolation and characterization

#### 2.4.1 Spirulina EV isolation

In order to isolate EVs, 2.5 L of axenic (cloned) *Spirulina* was harvested at OD_680_ = 1.3. Low‐velocity centrifugation steps (1000 × *g*, 2000 × *g*, 4000 × *g* and 10,000 × *g* for 11 min at 4°C) were performed to collect supernatant and to remove large cellular debris. The supernatant was filtered with a 0.45 µm filter system (Corning 430516 0.45 µm CA, low protein binding) and the flow‐through containing the EVs was concentrated approximately 10‐fold using Vivaspin 20, 100 kDa cut‐off filters (Sigma‐Aldrich) following the manufacturer's protocol to reach the practical volumes for ultracentrifugation. High‐velocity centrifugation (200,000 × *g* for 70 min at 3°C) was then performed on the concentrated supernatant using an Optima MAX‐XP with an MLA‐50 rotor and OPTISEAL tubes 361625 (Beckman Coulter). The supernatant was discarded carefully, and the pellet was resuspended in chilled PBS. The ultracentrifugation was repeated to ensure the soluble material was completely removed; the pellet (crude EVs) was resuspended in 2 mL chilled PBS (or in water, only for proteomics). The purified EVs were fractionated by size exclusion chromatography (SEC) using a qEVoriginal isolation column (Gen 2, 35 nm) mounted on an automatic fraction collector (AFC) machine (Izon) (Figure 1b). BCA protein assay (Pierce—Thermofisher) was performed to indirectly quantify the EV concentration by measuring EV surface proteins. We assessed the lipopolysaccharide (LPS) content of the SPEVs using 1 µL of the sample (equal to 5 × 10^8^ particles) and following the protocol provided by the kit manufacturer (A39552S, Pierce).

### 2.5 Orange EV isolation

Two Kg of oranges (*Citrus sinensis*) were washed twice with tap water and press‐juiced to produce ∼800 mL orange juice. The orange juice was passed through low‐velocity centrifugation steps (1000 × *g*, 2000 × *g* and 4000 × *g* for 10 min at 4°C) were performed to collect supernatant and to remove large cellular debris. The Supernatant was filtered with Whatman filter grade 1 and then a 0.45 PES (Corning) at 4°C. ∼180 mL filtered juice was ultracentrifuged (170,000 × *g* for 90 min at 4°C) using an Optima MAX‐XP with an MLA‐50 rotor and OPTISEAL tubes 361625 (Beckman Coulter) producing a pellet of EVs. The supernatant was discarded, and pellets were resuspended in 1 mL of 20 µM Tris‐HCl solution (Stanly et al., 2016) by vigorously vortexing for > 10 min. The resuspended EVs were combined and ultracentrifuged again with the same conditions, and the produced pellet was resuspended in PBS. The isolated orange EVs were fractionated by SEC using a qEVoriginal isolation column (Gen 2, 35 nm) mounted on an AFC machine (Izon).

### 2.6 EV size and concentration measurement

The size and concentration of EV fractions and combined FRC #2 and #3 EVs were measured using tuneable resistive pulse sensing (TRPS) and nanoparticle tracking analysis (NTA) methods.

NTA was performed using a NanoSight NS300 machine (Software v3.3/3.4.4; Malvern Pananalytical, Malvern, United Kingdom) with a 405 nm laser to assess EV size distribution and concentration. Triplicate, 1‐min videos were recorded, and data was presented as the mean of the three triplicates. The camera was set on level 10 with a detection threshold of 3. A syringe pump flow rate of 40 µL/min was used for each replicate.

TRPS was performed using an Izon Exoid and an NP100 nanopore. CPC100 calibration particles (1:1000 dilution) were used to calibrate the Nanopore. Measurements were accomplished with these Exoid settings: 850–1100 mV of voltage, 100–110 mA of current, 44.9–47 mm of stretch and 300–900 Pa for pressure.

### 2.7 Cryogenic transmission electron microscopy (cryo‐TEM)

EV samples were imaged by cryo‐TEM. Specimens were prepared using the Leica EM GP2 robotic vitrification system under controlled temperature (22°C) and relative humidity (95%). A 3 µL dispersion of EVs was applied onto a carbon‐coated perforated formvar film supported on a 200‐mesh copper TEM grid. Any excess solution was automatically blotted for a period of 3–3.5 s and swiftly plunged into liquid ethane near its freezing point (−180°C). The grids were stored under liquid nitrogen until imaging. The samples were imaged using a Jeol Cryo ARM 200 (JEM‐Z200FSC) transmission electron microscope (TEM) in a frozen hydrated state at −176°C. The microscope was equipped with a cold field emission gun (FEG) and an in‐column Omega energy filter. The images were captured with zero energy loss at an acceleration voltage of 200 kV and a filter setting of 20 eV. To ensure minimal exposure, the images were recorded under low‐dose conditions using the SerialEM software and a Gatan K2 direct detector camera (Mastronarde, 2005).

## 3 PROTEIN CONTENT CHARACTERIZATION

### 3.1 SDS‐PAGE gel and in‐gel digestion

Three replicates of 20 µg of SPEVs as measured by total protein measurement and a vehicle control, were resuspended in three parts water with one part 4 × reducing loading buffer (40% glycerol, 240 mM Tris‐HCL pH 6.8, 8% SDS, 0.04% Bromophenol blue, 5% beta‐mercaptoethanol,) and incubated at 95°C for 5 min before being loaded into each lane of a 12% sodium dodecyl sulphate polyacrylamide gel. Gels were run with a tris‐glycine 1% SDS buffer at 100 V for approximately 30 min, stained with 0.02% Coomassie blue for 10 min and de‐stained twice with 10% acetic acid and 50% methanol in water for 45 min. Each gel lane was excised and cut horizontally into four thin bands around 2 mm high and 10 mm wide. Similar‐sized gel bands from the vehicle control lane were excised. Each band was processed and analyzed downstream as an individual sample.

Excised bands were cut in half down the middle to improve the surface area and further de‐stained twice for 45 min at 37°C in 50% acetonitrile in 200 mM ammonium bicarbonate buffer (ambic). Gel bands were then dried in a vacuum centrifuge and reduced with 20 mM dithiothreitol in 25 mM ambic for 1 h at 65°C. Subsequently, gel bands were alkylated with 50 mM iodoacetamide in 25 mM ambic for 40 min in the dark at 37°C. Gel bands were soaked/submerged three times in 25 mM ambic for 15 min at 37°C then dried thoroughly again in a vacuum centrifuge. Each sample was then incubated for around 18 h at 37°C in 100 mM ambic containing 0.5 µg of Solu‐trypsin (Sigma‐Aldrich, USA). Digested peptides were extracted from gel bands by incubating slices in 50% acetonitrile and 0.1% trifluoroacetic acid (TFA) in water for 15 min at 37°C repeated three times. Extracted peptides were lyophilized before further downstream processing.

### 3.2 S‐trap proteomics preparation

S‐trap sample processing was used in addition to in‐gel digest to provide the opportunity for additional protein identification, as different proteomic processing methods might result in the enrichment of proteins with different properties. Three replicates of 32 µg SPEV as measured by total protein measurement were resuspended with 5% SDS in 50 mM triethylammonium bicarbonate (TEAB) and reduced at a final concentration of 20 mM tris (2‐carboxyethyl) phosphine hydrochloride for 15 min at 55°C. A vehicle control consisting of 5% SDS in 50 mM TEAB was also included and treated identically to the SPEVs. Samples were then alkylated for 10 min in the dark at room temperature at a final concentration of 40 mM iodoacetamide. Protein samples were acidified to a final concentration of approximately 2.5% phosphoric acid, precipitated with 90% methanol in 100 mM TEAB and bound onto S‐trap micro columns (Protifi, USA). Proteins bound to columns were washed with 90% methanol in 100 mM TEAB 3 times by centrifugation at 4000 × *g* and digested with 3.2 µg of Solu‐trypsin (Sigma‐Aldrich, USA) for around 18 h. Digested peptides were eluted from columns at 4000 × *g* with 50 mM TEAB, 0.2% formic acid then 50% acetonitrile in sequence and lyophilized before further downstream processing.

### 3.3 Solid‐phase sample clean‐up

All mass spectrometry samples prepared with in‐gel digestion and S‐trap columns were further desalted by solid‐phase extraction to remove interfering contaminants before acquisition on the mass spectrometer. Dried tryptic peptides were resuspended in 0.2% TFA bound onto four layers of Empore Octadecyl C18 (Supelco/Sigma‐Aldrich, USA) punched out by syringe cannulas and inserted into 200 µL pipette tips, referred to here as ‘stage tips’. All stage tips were washed with methanol and 0.2% TFA before use and all centrifugation steps were performed at 1000 × *g* for 2–4 min. Bound peptides were washed three times with 0.2% TFA and eluted with 60% acetonitrile and 0.2% formic acid in water. Peptides were then dried in a vacuum centrifuge and resuspended in 0.1% formic acid (Solvent A) for mass spectrometry acquisition.

### 3.4 Data acquisition

All replicates and controls were acquired on a NanoLC 415 (Eksigent, USA) liquid chromatography system running Solvent A and 0.1% formic acid in 100% HPLC grade acetonitrile (Solvent B) connected to a TripleTOF 6600 (AB Sciex, USA) mass spectrometer on data‐dependent acquisition (DDA) mode. Peptides from all replicates from the in‐gel and s‐trap method were respectively loaded on a Protocol C18 G (Trajan, Australia) or a C18 micro trap positive (Phenomenex, USA) 10 × 0.3 mm trap column with 10 µL/minute of Solvent A for 5 min. Peptides were then separated on a Proteocol C18 G203 3 um 200A 250 ´ 0.3 mm, column (Trajan, Australia) over a linear gradient of 3%–40% solvent B for 90 min at 5 µL/minute. Experimental parameters were: curtain gas = 35, ion source gas 1 = 25, ion source gas 2 = 30, ion spray voltage floating = 5000, turboheater temperature = 300 and declustering potential = 80. Ions were analysed with a TOF MS accumulation time of 249.9 ms and a collision energy of 10 across a mass window of 400–1250 followed by 31 experiments of 50 ms data‐dependent scans on product ions with a charge state between 2 and 5, a collision energy spread of 5 and a mass window of 100–1500.

### 3.5 Database search and protein identification

In order to process raw data files into identified proteins, acquired mass spectrometry data files from the In‐gel digest (one band from one replicate excluded due to acquisition fault) and S‐trap digest pipelines were analyzed separately. All searches were conducted against a combined protein database with 8701 unique sequences retrieved from Uniprot with 6050 and 5505 sequences from *Arthrospira platensis* (UP001059696) and from *Limnospira maxima* (UP000004061), respectively, and the removal of 2854 duplicate sequences. The protein database was further appended with 381 sequences from a universal protein contaminant library to rule out falsely identified peptides attributable to common laboratory contaminants. (Frankenfield et al., 2022) An iRT (Indexed retention time) peptide mixture (Biognosys, Switzerland) was added to the resuspension buffer and included in the database but not used for further analysis. Data files for all SPEV gel bands and replicates were pooled and searched against the library using the Paragon method on the ProteinPilot 5.0.1 software (AB Sciex, USA) with the following settings: sample type = identification; cys alkylation = iodoacetamide, with other cys mods possible; digestion = trypsin; instrument = TripleTOF 6600; Special factors = Gel based ID; focus = biological modifications; search effort = thorough ID; detected protein threshold (‘unused protein score’) ≥ 1.3 (95.0%); and run false‐discovery rate analysis = yes. Data files for all three SPEV S‐trap prepared replicates were searched with the same parameters except with the field ‘Gel‐based ID’ unchecked. Results from both In‐gel and S‐trap pipelines were combined and duplicate proteins were removed. Proteins with less than two peptides detected were also excluded from further analysis. Proteins were sorted by the average ‘unused protein score’ of detected proteins from the In‐gel digest and S‐trap digest pipeline each. ‘Unused proteins score’ was derived from the protein identification algorithm which is an indicator of the extent and strength of protein identification evidence unique to a particular protein.

### 3.6 Bioinformatics analyses of proteins

To infer and assign functional information about identified sequences, gene ontology (GO) annotation and analyses were performed on the Blast2GO software v6.0.3 (Biobam, Spain) (Götz et al., 2008) with the built‐in BLAST searching against the non‐redundant National Center for Biotechnology Information (NCBI) database (Sayers et al., 2023). BLAST searches were performed under the following settings: BLAST program = blastp‐fast, BLAST DB = nr, BLAST expectation value = 1.0E‐3, word size of 3 and a high‐scoring segment pair length cutoff value of 33. The REVIGO web application (Supek et al., 2011) was used to plot term similarity, Blast2GO node scores and the number of annotations for the respective term in the entire UniProt‐to‐GO database (Huntley et al., 2015) after filtering out redundant parent groups and terms containing less than 2 sequences. Analysis of protein families (Pfam) was performed on the InterPro web application (https://www.ebi.ac.uk/interpro/search/sequence/) (Paysan‐Lafosse et al., 2023) and the number of Pfam terms was quantified and cross‐referenced to identified proteins from the resulting output.

### 3.7 Immune response and adjuvanticity

#### 3.7.1 Experimental animals

Male and female C57BL/6 mice were bred at James Cook University (Townsville, Australia) and BALB/c mice aged 6–10 weeks and 25–30 g body weight were purchased from Ozgene Animal Resource Centre (Australia). Sample size *n* ≥ 3 per group was calculated as sufficient to attain statistical power based on previous studies (Gowthaman et al., 2019; Sikder et al., 2020). Mice were maintained in standard cages in a 12‐h light‐dark cycle, and commercial standard mouse pellet feed and water were provided ad libitum. The experiments were carried out at James Cook University Cairns Campus under Animal Ethics Approval (#A2777) and the National Health and Medical Research Council's Australian Code of Practice for the Care and Use of Animals for Scientific Purposes was followed.

#### 3.7.2 Experimental design and sample collection

C57BL/6 mice (*n* = 3) were injected intraperitoneally (i.p.) with 20 µg SPEVs per mouse in 100 µL PBS at days 0 and 1 and the animals were euthanized at day 2 to observe short‐term immune response (Figure 6a). In a separate experiment, BALB/c mice (*n* = 3) were injected i.p. with 20 µg (equivalent to 9.0 × 10^10^ particles of) SPEVs per mouse in PBS at day 0, 3, 6 and 9 and the mice were euthanized at day 10 to demonstrate long‐term immune response (Figure 6b). The control mice (*n* = 3) were injected i.p. with the same volume of PBS only. Peritoneal exudate cells were collected by i.p. injection of 5 mL ice‐cold PBS using a 5 mL syringe fitted with a 21‐gauge needle. The spleens were removed from mice and crushed using frost slides (CLS2948, Sigma) and hemolyzed by adding RBC lysis buffer (R7757, Sigma) for 3 min to isolate single cells. Live cells were counted using the trypan blue exclusion method.

To demonstrate the adjuvanticity of SPEVs, we used *Schistosoma haematobium* tetraspanin‐2 (*Sh*‐TSP‐2) expressed in *E. coli* and purified by immobilized metal ion affinity chromatography as a vaccine antigen, which has previously been reported as immunogenic by our group (Pearson et al. 2021). Male and female BALB/c mice (*n* = 5 per group except PBS group *n* = 4 and *Sh*‐TSP‐2 only *n* = 3) were immunized with 25 µg *Sh*‐TSP‐2 mixed with 25 µg (equivalent to 1.12 × 10^11^ particles of) SPEVs per mouse in PBS subcutaneously (sc) in the back region at days 0, 14 and 28 (Figure 6c). Positive control mice were injected with *Sh*‐TSP‐2 mixed with 25 µL 2% colloidal suspension of aluminium hydrogel (vac‐alu‐10, InvivoGen) and 5 µg of CpG oligonucleotide (tlrl‐1826, InvivoGen). The negative control groups were immunized with either *Sh*‐TSP‐2 only or PBS+alum/CpG or *Sh*‐TSP2+25 µg Orange EVs per mouse. The Orange EVs were used as a control for SPEVs to determine if EVs from a different source exhibit similar adjuvant effects. The mice were euthanized on day 40. After opening the thorax, blood samples were collected directly from the heart using an insulin syringe (Livingstone). Serum samples were obtained by centrifuging the blood at 10,000 RPM for 10 min. Peritoneal exudate cells were collected by injecting 3–5 mL of ice‐cold PBS into the peritoneal cavity of mice using a 19‐gauge needle attached to a 5 mL syringe, followed by centrifugation at 500G for 5 min. Spleen samples were collected, and single cells were isolated by crushing the spleen with frosted slides. Red blood cells (RBCs) were lysed using RBC lysis buffer (Sigma) for 3 min, followed by washing with FACs buffer and centrifugation at 500G for 5 min.

### 3.8 Staining of immune cells and flow cytometry

Immediately after single cell isolation, approximately 10^6^ cells were stained with 20 µL of anti‐cytokine antibody mix containing Near‐Infrared (NIR) Live‐Dead (ThermoFisher), BV650‐conjugated anti‐CD11b (M1/70, BioLegend), BV786‐conjugated anti‐CD11c (N418, BioLegend), BUV496‐conjugated anti‐F4/80 (T45‐2342, BD Bioscience), BUV421‐conjugated anti‐CD206 (C068C2, BioLegend), PE‐conjugated anti‐CD103 (M290, BD Bioscience), BUV395‐conjugated anti‐SiglecF (E50‐2440, BD Bioscience), AF700‐conjugated anti‐Ly6G (1A8, BD Bioscience) for 25 min at room temperature in the dark to stain the innate immune cells. To reduce non‐specific binding anti‐mouse CD16/32 (93, BioLegend), an Fc block was added to the staining panel.

For transcription factor staining, cells in separate panels were stained with 20 µL of anti‐cytokine antibody mix containing NIR Live‐Dead, Fc block and BV421‐conjugated anti‐CD25 (7D4, BD Bioscience) for 30 min at 4°C in the dark. Cells were then fixed and permeabilized with 100 µL eBioscience FoxP3/Transcription Factor Staining Kit (Thermo Fisher) for 20 min at 4°C in the dark. Samples were then stained with BV510‐conjugated anti‐CD4 (RM4‐5, BioLegend), FITC‐conjugated anti‐TCR (H57‐597, BioLegend), APC‐conjugated anti‐FoxP3 (150D/E4, eBioscience), PerCP.Cy5‐conjugated anti‐GATA3 (TWAJ, eBioscience), BV785‐conjugated anti‐Tbet (4B10, BioLegend) and PE‐conjugated anti‐RoRγt (AFKJS‐9, eBioscience) for 45 min at room temperature in the dark to identify regulatory T cell subsets.

To measure T cell intracellular cytokine production ex vivo, about 10^6^ cells were resuspended in 100 µL IMDM supplemented with 10% FBS, 100 U/mL penicillin, 100 µg/mL streptomycin, 2 mM L‐glutamine, 55 µM/L 2‐mercaptoethanol and 50 ng/mL phorbol 12‐myristate 13‐acetate (PMA) for cell stimulation and 1 µg/mL ionomycin as calcium ion transporter (Sigma). After 1 h incubation at 37°C with 5% CO_2_, GolgiPlug (100×, BD Bioscience) was added as a protein transport inhibitor and incubated for a further 3 h. Cells were washed and stained with NIR Live‐Dead and Fc block for 25 min at 4°C dark, followed by fixation and permeabilization for intracellular cytokine staining using 50 µL BD Cytofix/Cytoperm (BD Bioscience) for 20 min at room temperature. Cells were then stained with 20 µL of anti‐cytokine antibody mix containing PerCP.Cy5‐conjugated anti‐CD3 (17A2, BioLegend), BV605‐conjugated anti‐CD4 (RM4‐5, eBioscience), BUV496‐conjugated anti‐CD44 (IM7, BD Bioscience), PE.Cy7‐conjugated anti‐IFNγ (XMG1.2, BioLegend), FITC‐conjugated anti‐IL4 (11B11, eBioscience), BV421‐conjugated anti‐IL17 (eBio17B7, eBioscience) at 4°C in the dark overnight. The samples were acquired on a BD LSR FORTESSA with FACS‐Diva software (BD Bioscience). Data analysis was conducted using FlowJo software (Treestar).

### 3.9 THP‐1 cell culture

THP‐1 human monocyte cell line (88081201, Sigma) passaged up to 25 times was incubated with 200 nM PMA in RPMI with 10% FBS, 100 U/mL penicillin, 100 µg/mL streptomycin, 2 mM L‐glutamine, 55 µM/L 2‐mercaptoethanol (Sigma) at a concentration of 50,000 cells/well in a 96F microplate for 48 h to differentiate to macrophage cells. Following 24 h PMA starvation, the cells were incubated with 20 ng/mL of human recombinant IFNγ (AF300‐02‐20, Capsugel) and 1 ng/mL of human recombinant TNF𝛼 (AF300‐01A‐50, Capsugel) for 4 h to induce macrophage polarization into M1 or inflammatory macrophages. To remove IFNγ and TNF𝛼 from the medium, the cells were washed four times with plain RPMI. The cells were then stimulated with 10 ng/mL LPS (L6529, Sigma) and treated with 10 µg/mL SPEVs or PBS or 10 µg/mL dexamethasone (positive control) (D2915, Sigma) overnight at 37°C with 5% CO_2_to observe effects of SPEVs on TNF𝛼 production. The cell supernatants were collected and used for TNF𝛼 detection by ELISA. The experiment was repeated a minimum of two times to validate the results.

### 3.10 ELISA for serum IgG and TNF𝛼 assessment

Blood was collected from mice before and after immunizations into clot‐activating tubes and left undisturbed for at least 30 min at room temperature before centrifuging at 10,000 g for 10 min. To measure anti‐*Sh*‐TSP‐2 IgG response in mice, 96F microtitre plates (M4561, Sigma) were coated with 100 µL/well of 1 µg/mL *Sh*‐TSP‐2 in carbonate‐bicarbonate coating buffer butter pH 9.6 overnight at 4°C. After 4 × wash with PBS + 0.05% Tween‐20, the coated plates were blocked with 200 µL PBS Tween‐20 + 1% BSA overnight at 37°C. Serum samples were added in duplicate at a dilution of 1:100 for baseline sera, PBS sera, *Sh*‐TSP2 only sera, *Sh*‐TSP2 + SPEVs sera and *Sh*‐TSP2 + Orange EVs sera and 1:800 for *Sh*‐TSP2 + alum/CpG sera into blocking buffer and made a 2‐fold dilutions across the plates. Only PBS was added for blank correction. After 2 h incubation at 37°C and 4 × wash, 100 µL of goat anti‐mouse IgG‐HRP (H+L, 115‐035‐003, Jackson ImmunoResearch) at 1:5000 dilution in PBST or rabbit anti‐mouse IgG2a‐HRP (61‐0220, Thermo Fisher) at 1:2000 dilution in PBST was added to each well and incubated at 37°C for 1 h. After washing, 100 µL ABTS (11684302001, Sigma) was added for 30 min before absorbance was measured at 405 nm using a spectrophotometer (BMG Labtech). Cut‐off was determined as the mean OD of pooled baseline sera + 3 standard deviations. For the detection of TNF𝛼 in THP‐1 cell culture supernatants, the ELISA procedure was followed as instructed (88‐7346‐88, Thermo Fisher).

### 3.11 Statistical analysis

Normal distribution of pooled data from immune cell flow cytometry and ELISA was assessed using GraphPad Prism 10. All data from the experimental and control groups passed the D'Agostino‐Pearson omnibus normality test and, therefore, were compared using an unpaired *t*‐test. *p*‐values of ≤ 0.05 were considered significant.

## 4 RESULTS

### 4.1 Phylogenetic analysis of the Spirulina species

In order to identify the species of our Spirulina culture stock, we performed a phylogenetic analysis. Using the designed primers, MFS‐P1 and MFS‐P2, PCR for the partial 16SrRNA fragment amplified the predicted band of ∼1500 bp (Figure S1A). Nucleotide BLAST of the sequenced fragment revealed that our *Spirulina* stock is closely related to the three known *Spirulina* species, *Limnospira maxima*, *Limnospira fusiformis* (formerly known as *Spirulina maxima* and *Spirulina fusiformis*) and *Arthrospira platensis* (formerly known as *Spirulina platensis*) (Figure S1B). Calculated pairwise distance using the Maximum Composite Likelihood model showed no distance (0.000) between the query sequence and *L. maxima* and *L. fusiformis* (i.e., maximum similarity). However, the calculated distance between the query and *A. platensis* was 0.0143 (Table S1). Maximum Likelihood Tree following the Tamura‐Nei model (Tamura & Nei, 1993) illustrates the phylogenetic distance of the query sequence with the three closely related species, *L. maxima*, *L. fusiformis* and *A. platensis* (Figure S1C).

### 4.2 Cryo‐TEM detected OMVs and OIMVs of various sizes in the purified *Spirulina* EV sample

**FIGURE 2:**
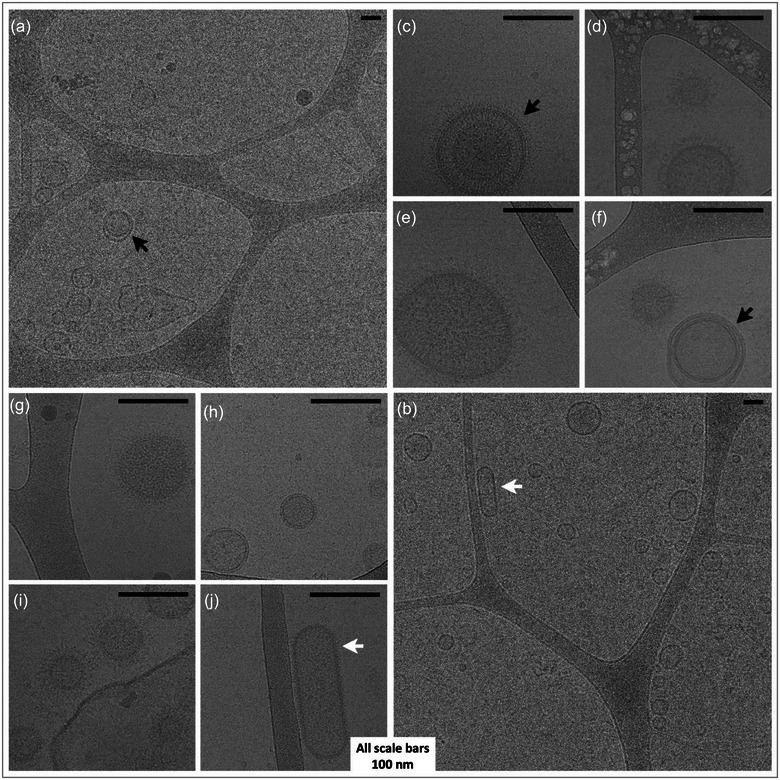
Cryogenic transmission electron micrographs of *Spirulina* extracellular vesicles (a) and (b). Wide‐field CryoTEM images (6000 × magnification) showcase SPEVs, specifically OMVs enclosed with a single bilayer phospholipid membrane, along with OIMVs indicated by black arrows, featuring a distinctive double bilayer phospholipid membrane (c—j). Narrow‐field CryoTEM images (60,000 × magnification) provide detailed views of SPEVs, revealing a range of sizes, predominantly comprising OMVs and a smaller fraction of OIMVs (black arrows). The white arrows indicate non‐spherical EVs. EV, extracellular vesicle; OMIV, outer‐inner membrane vesicles; OMV, Outer membrane vesicles; SPEV, *Spirulina* EVs.

To visualize the EVs and observe their size, shape and structure, we performed Cryo‐TEM. Wide‐view Cryo‐TEM images at 6000 × magnification confirmed the existence of EVs with spherical shapes and defining membranes of various sizes, referred to as OMVs. A minority of EVs were enclosed within two distinct layers of membranes, which is a characteristic of OIMVs. Non‐spherical membrane shapes, for example, rod or cylinder shapes, were also found, albeit less frequently (Figure 2a, b). At 60,000 × magnification, it was revealed that the majority of the EVs had a dense corona (i.e., peripheral filament‐like structures) with a visible length of up to 16.7 nm (Figure 2c−j); however, seldomly, we also found vesicles with no apparent corona (Figure 2f, h).

### 4.3 Purified SPEV concentration and size distribution

**FIGURE 3:**
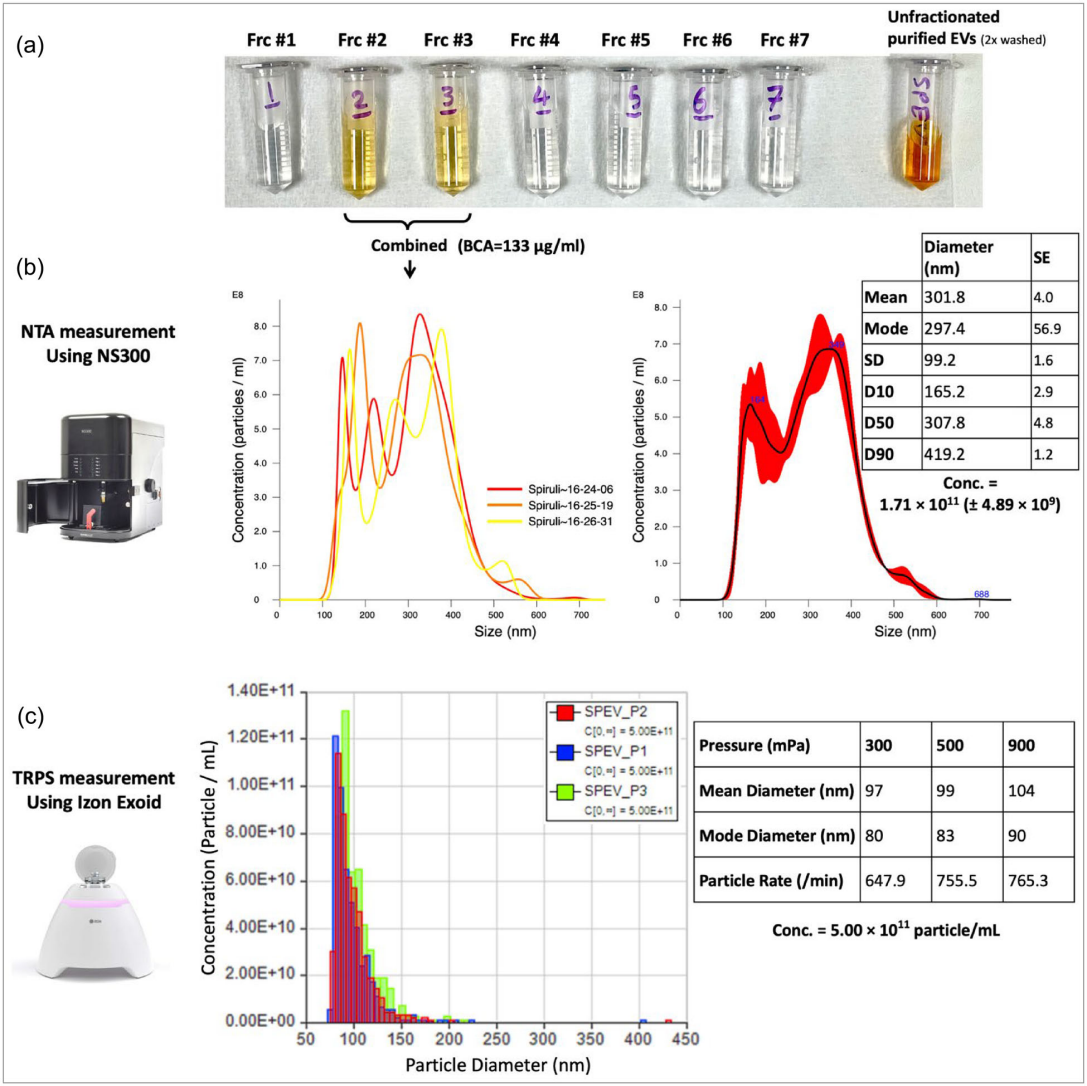
Size distribution and concentration of the isolated SPEV using two different technologies (a) Unfractionated SPEV suspension versus fractionated EVs (fractions 1–7) (b) Results for size distribution and concentration of SPEVs using NTA technology (c) Results for size distribution and concentration of SPEVs using TRPS technology. EV, extracellular vesicle; NTA, nanoparticle tracking analysis; SPEV, *Spirulina* EVs; TRPS, tuneable resistive pulse sensing.

To determine the size distribution and concentration of the purified EVs, we employed both NTA and tunable resistive pulse sensing (TRPS) methods. Double‐washed purified SPEVs (unfractionated) displayed a distinct dark orange hue. Following the SEC, visual inspection showed EVs were localized within fractions #2 and #3, both exhibiting a similar orange shade (Figure 3a). After combining the two fractions, the BCA protein assay calculated the surface protein of the crude EV suspension as 133 µg/mL.

The NTA analysis of three technical replicates for the SPEV suspension revealed a size distribution ranging from approximately 100 to 500 nm in diameter, with a mean diameter of 301.5 nm. The population displayed two peaks (bimodal) at 164 nm and 349 nm. The NTA results indicated a mean concentration of 1.71 × 10^11^ (± 4.89 × 10^9^) particles/mL (Figure 3b).

On the other hand, TRPS, employing an NP100 nanopore at three distinct pressure points, demonstrated a smaller size distribution range of 75 nm—225 nm, with a mean diameter of 100 nm. The concentration of EVs, as calculated by TRPS, was determined to be 5.00 × 10^11^ particles/mL (Figure 3c).

### 4.4 Orange EV concentration and size distribution

TRPS analysis of the purified orange EVs showed a concentration of 1.2 × 10^11^ particles/mL and a size distribution range of 50–450 nm in diameter, with a mean diameter of 113 nm (Figure S2A).

Cryo‐TEM micrographs of purified orange EVs at 6000 and 60,000 × magnifications demonstrated EVs with a range of ∼50‐500 nm in diameter (Figure S2B).

### 4.5 SPEV protein characterization

To further characterize the proteomic composition of isolated EVs, SPEV proteins from two separate pipelines were prepared, analyzed and identified using DDA mass spectrometry. A total of 54 unique protein sequences were identified from the combined results of both protein digestion and preparation methods. The most common description of proteins as identified via BLAST with the Blast2GO software was iron uptake porins, with a total of six sequences identified. Two sequences each were described as SdrD B‐like domain‐containing proteins, calcium‐binding proteins, AMIN domain‐containing proteins and carbon dioxide‐concentrating mechanism proteins (CcmK). Five sequences were described as hypothetical proteins. Of note, the three sequences with the highest ‘Unused protein score’ (ProteinPilot software score based on the confidence and uniqueness of corresponding peptide evidence) were all iron uptake porins (Table S2).

**FIGURE 4:**
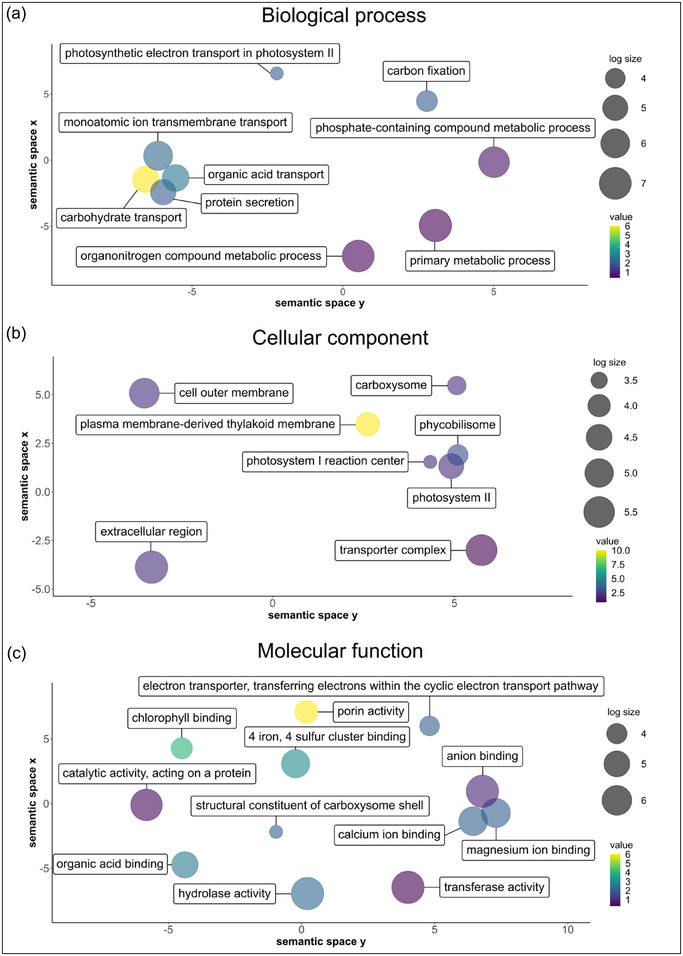
Sequences identified from SPEVs were analysed and annotated with Blast2GO to identify GO terms and quantify node scores. Results were summarised and visualised with ReviGO. Semantically similar GO terms will cluster more closely. The colour value indicates the Blast2GO node score. Circle size represents the Log10 number of annotations for a GO term in the entire Uniprot‐to‐GO database. EV, extracellular vesicle; GO, gene ontology; SPEV, *Spirulina* EVs.

A total of 30, 35 and 27 sequences were annotated with terms associated with the GO categories of ‘Biological process’, ‘Cellular component’ and ‘Molecular function’. GO results were filtered to remove redundant parent groups, GO terms with less than 2 sequences and visualized to show GO terms, ‘node score’ (Blast2GO software score based on the number of sequences weighted by the distance to the GO term) and annotation size for the GO term in the entire UniProt‐to‐GO database, where a large size indicates more annotations. The ‘biological process’ term with the highest node score was ‘carbohydrate transport’, as all sequences mapped to this GO term were the six same sequences described as iron transport porins (Figure 4a, Table S3). Other GO terms associated with the ‘transport’ parent GO term, such as ‘monoatomic ion transmembrane transport’ and ‘organic acid transport’, were also identified with relatively higher node scores (Figure 4a). Ten sequences were annotated with the ‘cellular component’ GO term ‘plasma membrane‐derived thylakoid membrane’. All three proteins under the GO term with the next highest node score, ‘phycobilisome’ were annotated as ‘plasma membrane‐derived thylakoid membrane’ proteins (Figure 4b, Table S3). Similarly, other identified sequences belonging to the thylakoid‐associated GO terms for ‘photosystem II’ or ‘photosystem I reaction center’ were also annotated as ‘plasma membrane‐derived thylakoid membrane’ proteins (Figure 4b, Table S3). Reflecting the aforementioned GO profiles, ‘porin activity’ had the highest node score amongst ‘molecular function’ GO terms as it was mapped to all six identified porins while the ‘chlorophyll binding’ and ‘4 iron, 4 sulphur cluster binding’ GO term displayed significant overlap with proteins associated with ‘plasma membrane‐derived thylakoid membrane’ (Figure 4c, Table S3).

**FIGURE 5:**
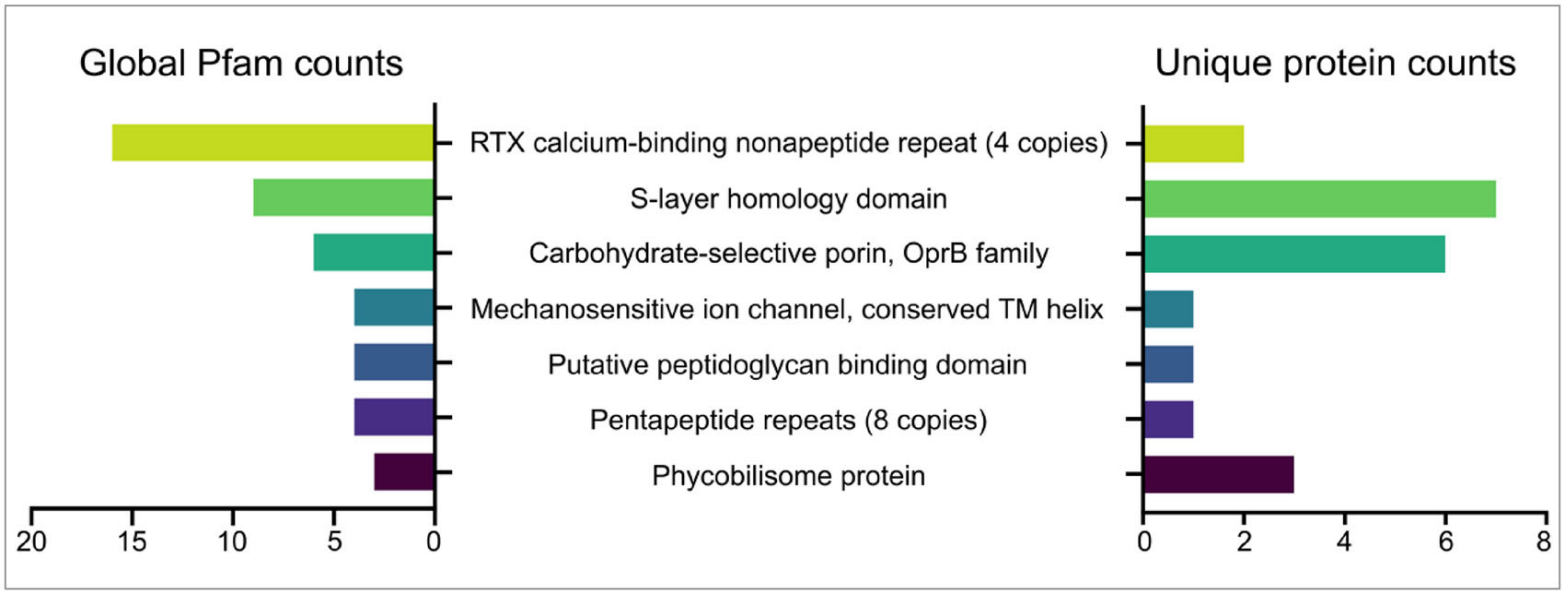
Sequences identified from SPEVs were analysed with InterPro to identify and quantify Pfam features. The top seven most common features across all sequences are displayed in the columns on the left, while the number of unique sequences with the corresponding Pfam feature is displayed on the right. SPEV, *Spirulina* EVs.

Pfam analysis showed that the seven most frequently identified protein domains and families across all proteins detected in SPEVs were ‘RTX calcium‐binding nonapeptide repeat (four copies)’, ‘S‐layer homology domain’, ‘Carbohydrate‐selective porin, OprB family’, ‘Mechanosensitive ion channel’, ‘conserved TM helix’, ‘Putative peptidoglycan binding domain’, ‘Pentapeptide repeats (8 copies)’ and ‘Phycobilisome protein’ (Figure 5). However, because multiple features can be found within one protein, we further enumerated the number of proteins that contained at least one of these top seven features (Figure 5). All six porins were identified to be part of the ‘Carbohydrate‐selective porin, OprB family’. These porins also incorporated one ‘S‐layer homology domain’ each, while another different protein described as a DUF1565 domain‐containing protein was found with three. In addition, three proteins were identified as ‘phycobilisome’ proteins, while all other Pfam terms were linked to only one or two sequences each across the proteome (Table S4).

### 4.6 Spirulina EVs induce inflammatory immune cell infiltration

**FIGURE 6:**
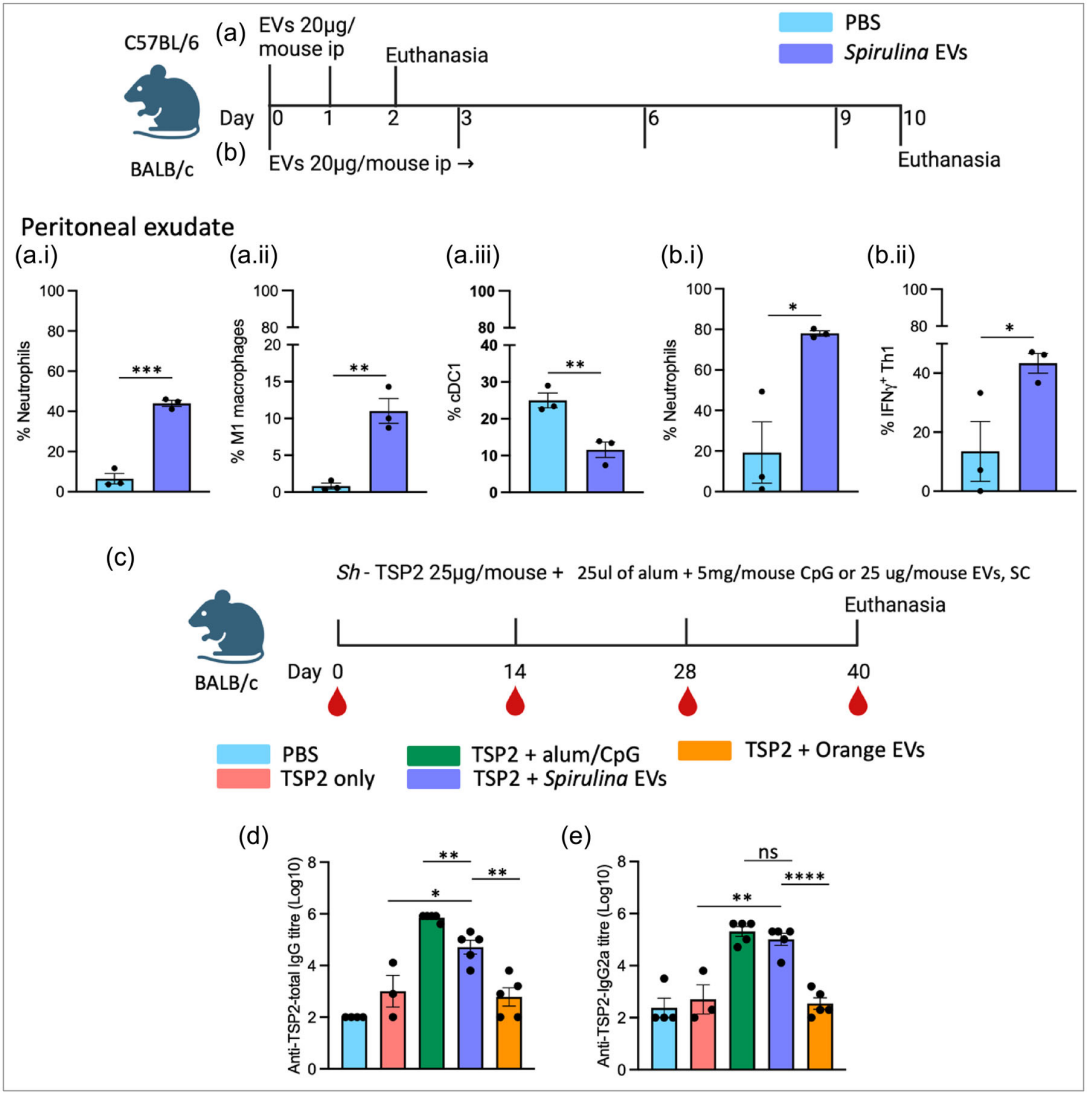
Spirulina EVs activate pro‐inflammatory cells and enhance anti‐TSP‐2 IgG production. Experimental design for short‐term (a) and long‐term immune response in mice (b). There was persistent neutrophilia (Live CD11b^+^ Ly6G^+^) (a.i and b.i), early higher M1 macrophage (Live CD11b^+^ F4/80^+^ CD11c^+^) (a.ii) and lower cDC1 (Live CD11c^+^ CD103^+^) (a.iii) infiltration and IFNγ^+^ Th1 cells (of Live CD3^+^ CD4^+^ CD44^+^) (b.ii) locally in the peritoneal cavity. Injection schedule for adjuvanticity experiment in BALB/c mice (c). TSP2 antigen‐specific total IgG (d) and IgG2a (e) response at Day 40. Error bars represent standard errors of the mean. Statistical analysis by T‐test, *p < 0.05, **p < 0.01, ***p < 0.001, ****p < 0.0001. EV, extracellular vesicle; TSP‐2, tetraspanin‐2.

Given that EVs play a role in cell‐to‐cell communication and influence cell function, we sought to identify which immune cells respond to SPEVs. To achieve this, we injected mice with 20 µg (150 µL) SPEVs intraperitoneally via two distinct regimens: (i) SPEVs were administered daily for two consecutive days to observe short‐term immune response in C57BL/6 mice or (ii) SPEVs were administered every third day for a total of four injections to demonstrate long‐term immune response in BALB/c mice. The selection of mouse strains for short‐term and long‐term immune response studies was based on strain availability, not to demonstrate variations between strains. Animals were euthanized 1 day after the last injection to observe short‐term (Day 2) and long‐term (Day 10) immune cell responses (Figure 6a, b). We collected peritoneal exudate cells to observe local responses and splenocytes to observe systemic immune responses. Interestingly, we observed persistent neutrophilia (Figure 6a.i, b.i), an early high percentage of M1 macrophages (Figure 6a.ii) and a lower percentage of conventional type 1 dendritic cell (cDC1) infiltration (Figure 6a.iii) at the site of injection. Moreover, we observed a lower percentage of M1 macrophages in the spleen (Figure S3E). Percentage of total cDCs, M2 macrophages and eosinophils in the peritoneal cavity (Figure S3A–C) and unchanged neutrophils and eosinophils in the spleen (Figure S3D, F). Furthermore, we observed a significantly higher percentage of IFNγ producing Th1 cells in mice long‐term injected with SPEVs in the peritoneal cavity compared to PBS‐injected mice (Figure 6b.ii), which remained unchanged in the spleen (S3L). It is important to recognize that neutrophils, eosinophils, macrophages and dendritic cells are early responders in the innate immune response, followed by helper (Th) and regulatory (Treg) T cells in the adaptive immune response. Key pro‐inflammatory cells include neutrophils, M1 macrophages, cDC1 cells, Th1 and Th17 cells, while the primary anti‐inflammatory cells responsible for reducing inflammation are eosinophils, M2 macrophages, cDC2 cells, Th2 and Treg cells. We further analyzed additional subsets of helper T cells (Th2 and Th17) as well as regulatory T cells (Tbet+ Treg, GATA3+ Treg and RoRγt+ Treg) from the peritoneal cavity and spleen to gain deeper insights into the broader immune cell response; however, other than high Th17 response in the spleen, all other immune cells didn't reach a level of significance compared to PBS injected control mice (Figure S3G–K, data of Treg subsets are not shown). We tested SPEVs for LPS content and found it to be relatively high at 5.5 EU/mL (FDA‐approved maximum 0.15 EU/mouse). However, TNF𝛼 secretion by THP‐1 cells upon stimulation with SPEVs remained non‐significant (Figure S4), indicating that LPS levels were unlikely to have been sufficiently high to drive inflammatory cytokine production by innate cells via TLR‐4 engagement.

### 4.7 Spirulina EVs enhance IgG antibody generation

As SPEVs induced local proinflammatory cell infiltration, we hypothesized that increased M1 macrophage levels might enhance adaptive immune responses towards vaccines as the core concept of adjuvanted vaccination is that adjuvants promote the generation of antigen‐presentation and co‐stimulatory signals by activating antigen‐presenting cells (Zhao et al., 2023). To detect the adjuvanticity of SPEVs, we immunized BALB/c mice with a model vaccine antigen *Sh*‐TSP‐2 (25 µg/mouse) mixed with 25 µg (188 µL) SPEVs and measured serum anti‐*Sh*‐TSP‐2 total IgG and subclass IgG2a (Figure 6c–e). We detected significantly higher serum anti‐*Sh*‐TSP2 total IgG (Figure 6d) and serum anti‐*Sh*‐TSP2 total IgG2a (Figure 6e) in mice that received SPEV‐adjuvanted *Sh*‐TSP‐2 compared to mice that received unadjuvanted *Sh*‐TSP‐2 or *Sh*‐TSP‐2 adjuvanted with Orange EVs. Although anti‐*Sh*‐TSP‐2 total IgG was lower than the alum/CpG adjuvanted group, SPEVs induced comparable levels of anti‐*Sh*‐TSP‐2 IgG2a.

## 5 DISCUSSION

This study presents the first documented evidence of isolating EVs from the edible cyanobacteria, Spirulina, and its morphological and molecular characterization. We found that SPEVs trigger pro‐inflammatory responses through innate cells without inducing toxicity upon parenteral delivery to mice. Finally, we showed that this immunomodulatory property of SPEVs can be utilized as an adjuvant for subunit vaccines.

Spirulina is a recognized functional food, and the evidence of its therapeutic effects is growing rapidly (Lafarga et al., 2020; Ragaza et al., 2020). A number of species within the *Limnospira* and *Arthrospira* genera are widely known as Spirulina (Nowicka‐Krawczyk et al., 2019). Through phylogenetic analysis of the 16SrRNA region, we found that our Spirulina stock is closely related to the *Limnospira* genus, specifically *L. maxima* and *L. fusiformis* (Figure S1 and Table S1).

Only a handful of studies have been published on EVs from cyanobacteria (Biller et al., 2014; Brito et al., 2017; Oliveira et al., 2016; Yin et al., 2019). However, to date, no study has reported the isolation of EVs from Spirulina. We found that with a combination of low‐velocity centrifugations, filtration and ultracentrifugation steps, followed by SEC, isolating EVs from a Spirulina culture is feasible and easily reproducible. The dark orange hue of the purified SPEVs was notable, which is consistent with a previous study that isolated EVs from other cyanobacteria, *Prochlorococcus*, *Synechococcus* and *Synechocystis* (Biller et al., 2022). It has been suggested that the outer membrane of some cyanobacteria could contain carotenoids (Jurgens & Weckesser, 1985), which could explain the orange colour of the SPEVs.

In Cryo‐TEM images, we found that the majority of SPEVs adopted the form of OMVs with one distinct bilayered phospholipid membrane; however, OIMVs with two layers of membrane were also found. This finding is in concordance with EVs from Gram‐negative bacteria, where EVs can emerge by budding out from the outer membrane or outer and inner membranes together (Reimer et al., 2021). A hypothesis for Gram‐negative bacteria suggests that when the outer membrane dissociates from periplasmic peptidoglycans to form OMVs, hydrolases may weaken parts of the peptidoglycan chain, enabling the inner membrane to invade the periplasmic space, resulting in a protrusion that forms an OIMV with an envelope similar to that of the bacteria. It can also explain the presence of genetic material and cytoplasmic proteins in EVs, as the cargo of OIMVs originates from the cytoplasm (Lima et al., 2020; Toyofuku et al., 2018). We did not find previous studies that reported OIMVs in cyanobacteria.

We also found that the majority of SPEVs showed a dense corona around their surface with a visible length of up to ∼16.6 nm; however, EVs with less or no visible corona were also found. Further investigations are required to determine a more accurate length and other physical and chemical properties of the SPEV corona.

Previous work has shown that EVs of Gram‐negative bacteria range from 20 to 400 nm in diameter. We measured the concentration and size distribution of SPEVs using two different methods, TRPS and NTA, which surprisingly demonstrated substantially different results. TRPS measured a higher concentration of EVs (5 × 10^11^ particle/mL) compared to NTA (1.71 × 10^11^ particle/mL). In regard to size distribution, TRPS depicted a population of EVs ranging from 75 to 225 nm with a mean diameter of 100 nm. NTA, however, depicted a bimodal population ranging from 100 nm to 500 nm with a calculated mean diameter of 301.5 nm. Cryo‐TEM images confirmed the existence of EVs as small as ∼25 nm and as large as ∼300 nm, with the majority of them being between 100 and 200 nm in diameter (Figure 2), which is closer to the number and size range determined by TRPS.

Through comprehensive proteomic analyses we identified several proteins and pfam that contribute to the biological function of EVs and could be contributing to the observed immune modulatory effects. The GO terms for ‘carbohydrate transport’ and ‘porin activity’ were highly enriched relative to the rest of the dataset. The six proteins annotated with these GO terms were also identified by pfam analysis to belong to the ‘Carbohydrate‐selective porin, OprB family’ and to contain one ‘s‐layer homology domain’ each. Moreover, another DUFF1565 domain‐containing protein with no GO annotation was identified to contain the ‘S‐layer homology domain’. S‐layers consist of arrays of S‐layer proteins (SLPs) that contain S‐layer homology (SLH) domains and can be found on the surface of many bacteria and archaea (Bharat et al., 2021; Fagan & Fairweather, 2014).

Although SLPs are sequentially diverse, it has been noted that SLP arrays and anchoring segments likely exist in only a limited combination of structure types (Bharat et al., 2021). S‐layers are an important part of cell biology and can comprise between 5% and 10% of total cell protein (Etienne‐Toumelin et al., 1995). In addition, the carbohydrate‐selective porin, OprB is typically responsible for transporting sugars such as glucose, fructose, sorbitol or glycerol as well as other small molecules of similar molecular weight such as arginine and lysine (Van Den Berg, 2012; Wylie & Worobec, 1995). In cyanobacteria, SLH and OprB domain containing proteins were previously found to be associated with outer membrane stability and metal transport despite their annotation as a carbohydrate‐selective porin (Cardoso et al., 2021; Schätzle et al., 2021). SLPs have been shown to be a major contributor to the virulence of *Clostridium difficile* where they promote Th1 and Th17 responses via interaction with TLR4 and subsequent downstream upregulation of IFN‐γ and IL‐17 (Ausiello et al., 2006; Ryan et al., 2011). Furthermore, SLPs from *C. difficile* can also stimulate macrophage migration and phagocytosis (Collins et al., 2014).

In this study, five of the seven proteins determined to contain a SLH domain were ranked amongst the top 10 of all detected proteins by ‘unused protein score’, while the overall top three proteins were identified as both SLH domain containing and OprB proteins.

The only detected protein identified as a SLH domain containing protein but not an OprB protein was a DUF1565 protein of unknown function that had the 7th highest ‘unused protein score’ but had no annotated GO term. Not much is known about the precise function of the DUF1565 protein or domain but it has been described from different taxa of bacteria such as gloeobacterales, nostocaetales and leptospiraceae (Pretre et al., 2013; Vieira et al., 2007). A leptospiral lipoprotein containing a DUF1565 domain, LIC10365 was shown to upregulate surface expression of ICAM‐1 and E‐selectin in endothelial cells (Vieira et al., 2007). If DUF15565 is indeed implicated in the induction of ICAM‐1 and E‐selectin, it may explain the influx of inflammatory cells observed in our study, as ICAM‐1 and E‐selectin promote the recruitment and adhesion of neutrophils and macrophages to an affected site (Chen et al., 2022; Silva et al., 2018; Singh et al., 2023). These findings suggest that SLH‐domain containing porins or SLH‐domain/DUF1565 domain containing proteins present in the EVs could be a significant contributor to the observed immunomodulatory effects, but further validation is required considering the broader sequence diversity of these types of proteins.

Sixteen RTX calcium‐binding nonapeptide repeat (four copies) domains were identified across the entire dataset, but these domains were found in only two proteins annotated as calcium ion binding proteins. Many proteins containing these calcium‐binding repeats have been described as cytolytic toxins possessing activity associated with the formation of cation pore channels (Baumann, 2019). While some of these proteins were described as cytolytic to neutrophils, macrophages and other monocytes in general (Ristow & Welch, 2019; Satchell, 2011), the RTX domain itself mainly functions to translocate its payload and is not necessarily toxic (Chenal & Ladant, 2018). Indeed, detoxified recombinant RTX domain proteins carrying CD8+ or CD4+ T cell epitopes have been shown to generate a functionally robust Th1 response and high titers of IgG against their targets, even without adjuvants (Chenal & Ladant, 2018; Fayolle et al., 2010; Préville et al., 2005). It may be possible that these RTX domain containing proteins in SPEVs are enhancing the immune stimulation provided by other detected proteins or assisting in antigen presentation to boost measured IgG levels in the current study. However, due to the lower rank of these two proteins in terms of ‘unused protein score’, the extent of this domain's contribution to the observed Th1 response in this study remains to be investigated.

EVs have recently been shown to have high potential for therapeutic purposes either by naturally occurring bioactive compounds on their surface and in their cargo or as drug delivery vehicles through bioengineered EVs and synthetically loaded EVs (Wiklander et al., 2019). Most of the studies have focused on human or mammalian cell‐derived EVs; however, in recent years, there has been accumulating evidence on the therapeutic effects of EVs derived from other forms of life, such as helminths, plants, bacteria and fungi (Drurey & Maizels, 2021; Jahromi & Fuhrmann, 2021; Karamanidou & Tsouknidas, 2021; Nenciarini & Cavalieri, 2023; Pirolli et al., 2021).

In this study, we showed that SPEVs induced a pro‐inflammatory immune response in mice. EVs from bacteria and microalgae are reported to be uptaken by mammalian cells (Ñahui Palomino et al., 2021; Picciotto et al., 2022). Following any microbial invasion, neutrophils, macrophages and dendritic cells are the first immune cell responders to microbe‐associated molecular patterns (MAMPs). Consequently, IFNγ derived from type 1 T helper (Th1) cells is necessary to enhance macrophage infiltration and subsequent antigen presentation. We found that SPEVs induced neutrophilia and significantly higher type 1 macrophage (M1) infiltration at the site of injection and later IFNγ‐producing Th1 cell aggregation (Figure 6). A similar immune response mechanism exists when adjuvants such as complete Freund's adjuvant or Alum+CpG are used to effectively stimulate cell‐mediated and humoral immune responses to a wide variety of antigens (Leenaars et al., 1994; Sanchez et al., 1980; Yang et al., 2023). IFNγ serves to recruit and activate macrophages, promoting granuloma formation and the inflammatory process during bacterial infection (Flynn & Chan, 2001). A component of cyanobacteria EVs is LPS, which could represent MAMPs and induce a pro‐inflammatory response (Matinha‐Cardoso et al., 2022; Rybak & Robatzek, 2019). Although the LPS content of SPEVs used in our study was found to be relatively high, we observed no noticeable symptoms (fever, piloerection, inappetence, isolation, reduced movement) related to LPS‐induced inflammation following SPEV injection in mice in contrast to LPS from other Gram‐negative bacteria (Zhao et al., 2019). The role of SLPs and RTX of SPEVs in inducing pro‐inflammatory responses could be an area of further investigation.

In our study, we found SPEVs have no effect in reducing TNF𝛼 production in LPS‐induced inflammatory responses in THP‐1 cell derived macrophages (Figure S3). A similar finding was reported in a meta‐analysis study with *Spirulina* extracts (Mohiti et al., 2021). However, Nawrocka *et al.* (Nawrocka et al., 2017) found that *Spirulina platensis* water extracts effectively suppressed LPS‐induced inflammation in macrophages. Moreover, the authors found that horses fed with *Spirulina* in diet supplementation reduced body weight and improved insulin sensitivity correlated with reduced inflammation. The phycocyanin and β‐carotene components of *Spirulina* are reported to have anticancer, anti‐inflammatory and radical‐scavenging properties. Interestingly, the SPEVs in our study showed proinflammatory properties.

We then tested the pro‐inflammatory properties of SPEVs as an adjuvant in enhancing antigen‐specific antibody production. Vaccination of mice with *Sh*‐TSP‐2‐SPEVs induced comparable total IgG and IgG2a antibody levels to *Sh*‐TSP‐2 adjuvanted with alum/CpG, and these antibody levels were significantly higher than those of mice that received *Sh*‐TSP‐2 alone or *Sh*‐TSP‐2 with orange EVs (Figure 6d, e). This indicates that SPEVs contain molecules that can induce an adjuvant response to vaccine antigens in a safe manner. We exclude the possible role of LPS as the adjuvanting component because orange EVs contained a similar concentration of LPS but failed to enhance IgG responses to the vaccine antigen. Moreover, Spirulina LPS has been reported not to have pro‐inflammatory effects (Okuyama et al., 2017). Although alum is a widely used adjuvant for human vaccines, it sometimes fails to induce a robust cell‐mediated immune response and is not effective with some antigens such as typhoid vaccine, *Haemophilus influenzae* type b vaccine, which limits its utility (Gupta & Siber, 1995; Hogenesch, 2012).

Ultimately, SPEVs could be utilized in a two‐pronged vaccination strategy whereby *Spirulina* with endogenous adjuvant properties can be genetically modified to express a foreign vaccine antigen on its surface (Jiji et al., 2023).

In conclusion, our study isolated and characterized EVs from *Spirulina* cyanobacteria in a convenient and reproducible way for the first time. Proteomic characterization of SPEVs identified several potential immunomodulatory components, including SLP, SLH‐domain and RTX proteins, which might have contributed to the pro‐inflammatory responses in mice without significant clinical symptoms. Finally, we showed that the pro‐inflammatory properties of SPEVs can be utilized as an adjuvant for subunit vaccines, opening up new avenues of industrial biotechnology that exploit the biotherapeutic properties of this microalgae.

## AUTHOR CONTRIBUTIONS

**Mohammad Farouq Sharifpour**: Conceptualization (lead); data curation (equal); formal analysis (equal); investigation (equal); methodology (equal); project administration (lead); software (equal); validation (equal); visualization (equal); writing ‐ original draft (lead); writing ‐ review & editing (lead). **Suchandan Sikder**: Conceptualization (equal); data curation (equal); formal analysis (equal); investigation (equal); methodology (equal); software (equal); validation (equal); visualization (equal); writing ‐ original draft (equal); writing ‐ review & editing (equal). **Yide Wong**: Conceptualization (equal) data curation (equal); formal analysis (equal); investigation (equal); methodology (equal); software (equal); validation (equal); visualization (equal); writing ‐ original draft (equal); writing ‐ review & editing (equal). **Na'ama Koifman**: Formal analysis (supporting); investigation (supporting); methodology (supporting); writing ‐ review & editing (supporting). **Tamara Thomas**: Investigation (supporting). **Robert Courtney**: Investigation (supporting); writing ‐ review & editing (supporting). **Jamie Seymour**: Investigation (supporting); methodology (supporting); writing ‐ review & editing (supporting). **Alex Loukas**: Conceptualization (supporting); funding acquisition (lead); methodology (equal); project administration (equal); resources (lead); supervision (lead); validation (supporting); writing ‐ review & editing (equal). All authors have read and agreed to the published version of the manuscript.

## CONFLICT OF INTEREST STATEMENT

The authors declare no competing interests.

## Supporting information



Supporting Information

Supporting Information

Supporting Information

Supporting Information

## Data Availability

The mass spectrometry proteomics data have been deposited to the ProteomeXchange Consortium via the PRIDE (Perez‐Riverol et al., 2022) partner repository with the dataset identifier PXD052246.

## References

[jex270025-bib-0001] Adamo, G. , Fierli, D. , Romancino, D. P. , Picciotto, S. , Barone, M. E. , Aranyos, A. , Božič, D. , Morsbach, S. , Raccosta, S. , Stanly, C. , Paganini, C. , Gai, M. , Cusimano, A. , Martorana, V. , Noto, R. , Carrotta, R. , Librizzi, F. , Randazzo, L. , Parkes, R. , … Bongiovanni, A. (2021). Nanoalgosomes: Introducing extracellular vesicles produced by microalgae. Journal of Extracellular Vesicles, 10(6), e12081. 10.1002/jev2.12081 33936568 PMC8077145

[jex270025-bib-0002] Ausiello, C. M. , Cerquetti, M. , Fedele, G. , Spensieri, F. , Palazzo, R. , Nasso, M. , Frezza, S. , & Mastrantonio, P. (2006). Surface layer proteins from Clostridium difficile induce inflammatory and regulatory cytokines in human monocytes and dendritic cells. Microbes and Infection, 8(11), 2640–2646. 10.1016/j.micinf.2006.07.009 16935543

[jex270025-bib-0003] Baumann, U. (2019). Structure‐function relationships of the repeat domains of RTX toxins. Toxins, 11(11), 657. 10.3390/toxins11110657 31718085 PMC6891781

[jex270025-bib-0004] Bharat, T. A. M. , von Kügelgen, A. , & Alva, V. (2021). Molecular logic of prokaryotic surface layer structures. Trends in Microbiology, 29, 405–415. 33121898 10.1016/j.tim.2020.09.009 PMC8559796

[jex270025-bib-0005] Biller, S. J. , Muñoz‐Marín, M. D. C. , Lima, S. , Matinha‐Cardoso, J. , Tamagnini, P. , & Oliveira, P. (2022). Isolation and characterization of cyanobacterial extracellular vesicles. Journal of Visualized Experiments : JoVE, 00(180), 00–00. 10.3791/63481 35188125

[jex270025-bib-0006] Biller, S. J. , Schubotz, F. , Roggensack, S. E. , Thompson, A. W. , Summons, R. E. , & Chisholm, S. W. (2014). Bacterial vesicles in marine ecosystems. Science, 343(6167), 183–186. 10.1126/science.1243457 24408433

[jex270025-bib-0007] Brito, Â. , Ramos, V. , Mota, R. , Lima, S. , Santos, A. , Vieira, J. , Vieira, C. P. , Kaštovský, J. , Vasconcelos, V. M. , & Tamagnini, P. (2017). Description of new genera and species of marine cyanobacteria from the Portuguese Atlantic coast. Molecular Phylogenetics and Evolution, 111, 18–34. 10.1016/j.ympev.2017.03.006 28279808

[jex270025-bib-0008] Cardoso, D. , Lima, S. , Matinha‐Cardoso, J. , Tamagnini, P. , & Oliveira, P. (2021). The role of outer membrane protein(S) harboring SLH/OprB‐domains in extracellular vesicles’ production in *Synechocystis* sp. PCC 6803. Plants, 10, 2757. 34961227 10.3390/plants10122757 PMC8707739

[jex270025-bib-0009] Chen, W. , Zheng, W. , Liu, S. , Su, Q. , Ding, K. , Zhang, Z. , Luo, P. , Zhang, Y. , Xu, J. , Yu, C. , Li, W. , & Huang, Z. (2022). SRC‐3 deficiency prevents atherosclerosis development by decreasing endothelial ICAM‐1 expression to attenuate macrophage recruitment. International Journal of Biological Sciences, 18(15), 5978–5993. 10.7150/ijbs.74864 36263184 PMC9576506

[jex270025-bib-0010] Chen, Y. H. , Chang, G. K. , Kuo, S. M. , Huang, S. Y. , Hu, I. C. , Lo, Y. L. , & Shih, S. R. (2016). Well‐tolerated Spirulina extract inhibits influenza virus replication and reduces virus‐induced mortality. Scientific Reports, 6, 24253. 10.1038/srep24253 27067133 PMC4828654

[jex270025-bib-0011] Chenal, A. , & Ladant, D. (2018). Bioengineering of *Bordetella pertussis* adenylate cyclase toxin for antigen‐delivery and immunotherapy. Toxins, 10(7), 302. 10.3390/toxins10070302 30037010 PMC6070788

[jex270025-bib-0012] Collins, L. E. , Lynch, M. , Marszalowska, I. , Kristek, M. , Rochfort, K. , O'Connell, M. , Windle, H. , Kelleher, D. , & Loscher, C. E. (2014). Surface layer proteins isolated from Clostridium difficile induce clearance responses in macrophages. Microbes and Infection, 16(5), 391–400. 10.1016/j.micinf.2014.02.001 24560642

[jex270025-bib-0013] Dinicolantonio, J. J. , Bhat, A. G. , & Okeefe, J. (2020). Effects of spirulina on weight loss and blood lipids: A review. Open Heart, 7, e001003. 32201580 10.1136/openhrt-2018-001003 PMC7061888

[jex270025-bib-0014] Drurey, C. , & Maizels, R. M. (2021). Helminth extracellular vesicles: Interactions with the host immune system. Molecular Immunology, 137, 124–133. 34246032 10.1016/j.molimm.2021.06.017 PMC8636279

[jex270025-bib-0015] Etienne‐Toumelin, I. , Sirard, J. C. , Duflot, E. , Mock, M. , & Fouet, A. (1995). Characterization of the Bacillus anthracis S‐layer: Cloning and sequencing of the structural gene. Journal of Bacteriology, 177, 614–620. 7836294 10.1128/jb.177.3.614-620.1995 PMC176635

[jex270025-bib-0016] Fadeev, E. , Carpaneto Bastos, C. , Hennenfeind, J. H. , Biller, S. J. , Sher, D. , Wietz, M. , & Herndl, G. J. (2022). Characterization of membrane vesicles in *Alteromonas macleodii* indicates potential roles in their copiotrophic lifestyle. Microlife, 4, uqac025. 10.1093/femsml/uqac025 37223730 PMC10117737

[jex270025-bib-0017] Fagan, R. P. , & Fairweather, N. F. (2014). Biogenesis and functions of bacterial S‐layers. Nature Reviews. Microbiology, 12(3), 211–222. 10.1038/nrmicro3213 24509785

[jex270025-bib-0018] Fayolle, C. , Davi, M. , Dong, H. , Ritzel, D. , Le Page, A. , Knipping, F. , Majlessi, L. , Ladant, D. , & Leclerc, C. (2010). Induction of anti‐Tat neutralizing antibodies by the CyaA vector targeting dendritic cells: Influence of the insertion site and of the delivery of multicopies of the dominant Tat B‐cell epitope. Vaccine, 28(42), 6930–6941. 10.1016/j.vaccine.2010.07.059 20728521

[jex270025-bib-0019] Flynn, J. L. , & Chan, J. (2001). Immunology of tuberculosis. Annual Review of Immunology, 19, 93–129. 10.1146/annurev.immunol.19.1.93 11244032

[jex270025-bib-0020] Frankenfield, A. M. , Ni, J. , Ahmed, M. , & Hao, L. (2022). Protein contaminants matter: Building universal protein contaminant libraries for DDA and DIA proteomics. Journal of Proteome Research, 21, 2104–2113. 35793413 10.1021/acs.jproteome.2c00145 PMC10040255

[jex270025-bib-0021] Götz, S. , García‐Gómez, J. M. , Terol, J. , Williams, T. D. , Nagaraj, S. H. , Nueda, M. J. , Robles, M. , Talón, M. , Dopazo, J. , & Conesa, A. (2008). High‐throughput functional annotation and data mining with the Blast2GO suite. Nucleic Acids Research, 36(10), 3420–3435. 10.1093/nar/gkn176 18445632 PMC2425479

[jex270025-bib-0022] Gowthaman, U. , Chen, J. S. , Zhang, B. , Flynn, W. F. , Lu, Y. , Song, W. , Joseph, J. , Gertie, J. A. , Xu, L. , Collet, M. A. , Grassmann, J. D. S. , Simoneau, T. , Chiang, D. , Berin, M. C. , Craft, J. E. , Weinstein, J. S. , Williams, A. , & Eisenbarth, S. C. (2019). Identification of a T follicular helper cell subset that drives anaphylactic IgE. Science, 365(6456), eaaw6433. 10.1126/science.aaw6433 31371561 PMC6901029

[jex270025-bib-0023] Gupta, R. K. , & Siber, G. R. (1995). Adjuvants for human vaccines–current status, problems and future prospects. Vaccine, 13, 1263–1276. 8585280 10.1016/0264-410x(95)00011-o

[jex270025-bib-0024] Hogenesch, H. (2012). Mechanism of immunopotentiation and safety of aluminum adjuvants. Frontiers in immunology, 3, 406. 23335921 10.3389/fimmu.2012.00406 PMC3541479

[jex270025-bib-0025] Hu, Q. , Hu, Z. , Yan, X. , Lu, J. , & Wang, C. (2024). Extracellular vesicles involved in growth regulation and metabolic modulation in Haematococcus pluvialis. Biotechnology for Biofuels and Bioproducts, 17(1), 15. 10.1186/s13068-024-02462-z 38282041 PMC10823724

[jex270025-bib-0026] Hu, S. , Fan, X. , Qi, P. , & Zhang, X. (2019). Identification of anti‐diabetes peptides from Spirulina platensis. Journal of Functional Foods, 56, 333–341.

[jex270025-bib-0027] Huntley, R. P. , Sawford, T. , Mutowo‐Meullenet, P. , Shypitsyna, A. , Bonilla, C. , Martin, M. J. , & O'Donovan, C. (2015). The GOA database: Gene ontology annotation updates for 2015. Nucleic Acids Research, 43(Database issue), D1057–D1063. 10.1093/nar/gku1113 25378336 PMC4383930

[jex270025-bib-0028] Jahromi, L. P. , & Fuhrmann, G. (2021). Bacterial extracellular vesicles: Understanding biology promotes applications as nanopharmaceuticals. Advanced Drug Delivery Reviews, 173, 125–140. 33774113 10.1016/j.addr.2021.03.012

[jex270025-bib-0029] Jiji, M. G. , Ninan, M. A. , Thomas, V. P. , & Thomas, B. T. (2023). Edible microalgae: Potential candidate for developing edible vaccines. Vegetos (Bareilly, India), 1–6. *Advance online publication*. 10.1007/s42535-023-00636-y PMC10136395 37359124

[jex270025-bib-0030] Jurgens, U. J. , & Weckesser, J. (1985). Carotenoid‐containing outer membrane of Synechocystis sp. strain PCC6714. Journal of Bacteriology, 164, 384–389. 3930470 10.1128/jb.164.1.384-389.1985 PMC214255

[jex270025-bib-0031] Karamanidou, T. , & Tsouknidas, A. (2021). Plant‐derived extracellular vesicles as therapeutic nanocarriers. International Journal of Molecular Sciences, 23(1), 191. 10.3390/ijms23010191 35008617 PMC8745116

[jex270025-bib-0032] Kulshreshtha, A. , Zacharia, A. J. , Jarouliya, U. , Bhadauriya, P. , Prasad, G. B. , & Bisen, P. S. (2008). Spirulina in health care management. Current Pharmaceutical Biotechnology, 9(5), 400–405. 10.2174/138920108785915111 18855693

[jex270025-bib-0033] Lafarga, T. , Fernández‐Sevilla, J. M. , González‐López, C. , & Acién‐Fernández, F. G. (2020). Spirulina for the food and functional food industries. Food Research International, 137, 109356. 33233059 10.1016/j.foodres.2020.109356

[jex270025-bib-0034] Leenaars, P. P. , Hendriksen, C. F. , Angulo, A. F. , Koedam, M. A. , & Claassen, E. (1994). Evaluation of several adjuvants as alternatives to the use of Freund's adjuvant in rabbits. Veterinary Immunology and Immunopathology, 40, 225–241. 8160361 10.1016/0165-2427(94)90022-1

[jex270025-bib-0035] Lima, S. , Matinha‐Cardoso, J. , Tamagnini, P. , & Oliveira, P. (2020). Extracellular vesicles: An overlooked secretion system in cyanobacteria. Life, 10, 129. 32751844 10.3390/life10080129 PMC7459746

[jex270025-bib-0036] Martínez‐Sámano, J. , Torres‐Montes de Oca, A. , Luqueño‐Bocardo, O. I. , Torres‐Durán, P. V. , & Juárez‐Oropeza, M. A. (2018). Spirulina maxima decreases endothelial damage and oxidative stress indicators in patients with systemic arterial hypertension: results from exploratory controlled clinical trial. Marine Drugs, 16, 496. 30544795 10.3390/md16120496 PMC6315658

[jex270025-bib-0037] Mastronarde, D. N. (2005). Automated electron microscope tomography using robust prediction of specimen movements. Journal of Structural Biology, 152, 36–51. 16182563 10.1016/j.jsb.2005.07.007

[jex270025-bib-0038] Matinha‐Cardoso, J. , Coutinho, F. , Lima, S. , Eufrásio, A. , Carvalho, A. P. , Oliva‐Teles, A. , Bessa, J. , Tamagnini, P. , Serra, C. R. , & Oliveira, P. (2022). Novel protein carrier system based on cyanobacterial nano‐sized extracellular vesicles for application in fish. Microbial Biotechnology, 15(8), 2191–2207. 10.1111/1751-7915.14057 35419949 PMC9328742

[jex270025-bib-0039] Mohiti, S. , Zarezadeh, M. , Naeini, F. , Tutunchi, H. , Ostadrahimi, A. , Ghoreishi, Z. , & Ebrahimi Mamaghani, M. (2021). Spirulina supplementation and oxidative stress and pro‐inflammatory biomarkers: A systematic review and meta‐analysis of controlled clinical trials. Clinical and Experimental Pharmacology & Physiology, 48(8), 1059–1069. 10.1111/1440-1681.13510 33908048

[jex270025-bib-0040] Morist, A. , Montesinos, J. L. , Cusidó, J. A. , & Gòdia, F. (2001). Recovery and treatment of Spirulina platensis cells cultured in a continuous photobioreactor to be used as food. Process Biochemistry, 37, 535–547.

[jex270025-bib-0041] Ñahui Palomino, R. A. , Vanpouille, C. , Costantini, P. E. , & Margolis, L. (2021). Microbiota–host communications: Bacterial extracellular vesicles as a common language. Plos Pathogens, 17, e1009508. 33984071 10.1371/journal.ppat.1009508 PMC8118305

[jex270025-bib-0042] Nawrocka, D. , Kornicka, K. , Śmieszek, A. , & Marycz, K. (2017). Spirulina platensis improves mitochondrial function impaired by elevated oxidative stress in adipose‐derived mesenchymal stromal cells (ASCs) and intestinal epithelial cells (IECs), and enhances insulin sensitivity in equine metabolic syndrome (EMS) horses. Marine Drugs, 15(8), 237. 10.3390/md15080237 28771165 PMC5577592

[jex270025-bib-0043] Nenciarini, S. , & Cavalieri, D. (2023). Immunomodulatory potential of fungal extracellular vesicles: Insights for therapeutic applications. Biomolecules, 13, 1487. 37892168 10.3390/biom13101487 PMC10605264

[jex270025-bib-0044] Nowicka‐Krawczyk, P. , Mühlsteinová, R. , & Hauer, T. (2019). Detailed characterization of the Arthrospira type species separating commercially grown taxa into the new genus Limnospira (Cyanobacteria). Scientific Reports, 9(1), 694. 30679537 10.1038/s41598-018-36831-0 PMC6345927

[jex270025-bib-0045] Okuyama, H. , Tominaga, A. , Fukuoka, S. , Taguchi, T. , Kusumoto, Y. , & Ono, S. (2017). Spirulina lipopolysaccharides inhibit tumor growth in a Toll‐like receptor 4‐dependent manner by altering the cytokine milieu from interleukin‐17/interleukin‐23 to interferon‐γ. Oncology Reports, 37(2), 684–694. 10.3892/or.2017.5346 28075473 PMC5355664

[jex270025-bib-0046] Oliveira, P. , Martins, N. M. , Santos, M. , Pinto, F. , Büttel, Z. , Couto, N. A. , Wright, P. C. , & Tamagnini, P. (2016). The versatile TolC‐like Slr1270 in the cyanobacterium Synechocystis sp. PCC 6803. Environmental Microbiology, 18(2), 486–502. 10.1111/1462-2920.13172 26663346

[jex270025-bib-0047] Paysan‐Lafosse, T. , Blum, M. , Chuguransky, S. , Grego, T. , Pinto, B. L. , Salazar, G. A. , Bileschi, M. L. , Bork, P. , Bridge, A. , Colwell, L. , Gough, J. , Haft, D. H. , Letunić, I. , Marchler‐Bauer, A. , Mi, H. , Natale, D. A. , Orengo, C. A. , Pandurangan, A. P. , Rivoire, C. , … Bateman, A. (2023). InterPro in 2022. Nucleic Acids Research, 51(D1), D418–D427. 10.1093/nar/gkac993 36350672 PMC9825450

[jex270025-bib-0047a] Pearson, M. S. , Tedla, B. A. , Mekonnen, G. G. , Proietti, C. , Becker, L. , Nakajima, R. , Jasinskas, A. , Doolan, D. L. , Amoah, A. S. , Knopp, S. , Rollinson, D. , Ali, S. M. , Kabole, F. , Hokke, C. H. , Adegnika, A. A. , Field, M. A. , van Dam, G. , Corstjens, P. L. A. M. , Mduluza, T. , … Loukas, A. (2021). Immunomics‐guided discovery of serum and urine antibodies for diagnosing urogenital schistosomiasis: a biomarker identification study. The Lancet. Microbe, 2(11), e617–e626. 10.1016/S2666-5247(21)00150-6 34977830 PMC8683377

[jex270025-bib-0048] Perez‐Riverol, Y. , Bai, J. , Bandla, C. , García‐Seisdedos, D. , Hewapathirana, S. , Kamatchinathan, S. , Kundu, D. J. , Prakash, A. , Frericks‐Zipper, A. , Eisenacher, M. , Walzer, M. , Wang, S. , Brazma, A. , & Vizcaíno, J. A. (2022). The PRIDE database resources in 2022: A hub for mass spectrometry‐based proteomics evidences. Nucleic Acids Research, 50(D1), D543–D552. 10.1093/nar/gkab1038 34723319 PMC8728295

[jex270025-bib-0049] Picciotto, S. , Barone, M. E. , Fierli, D. , Aranyos, A. , Adamo, G. , BoŽič, D. , Romancino, D. P. , Stanly, C. , Parkes, R. , Morsbach, S. , Raccosta, S. , Paganini, C. , Cusimano, A. , Martorana, V. , Noto, R. , Carrotta, R. , Librizzi, F. , Capasso Palmiero, U. , Santonicola, P. , … Bongiovanni, A. (2021). Isolation of extracellular vesicles from microalgae: Towards the production of sustainable and natural nanocarriers of bioactive compounds. Biomaterials Science, 9(8), 2917–2930. 10.1039/d0bm01696a 33620041

[jex270025-bib-0050] Picciotto, S. , Santonicola, P. , Paterna, A. , Rao, E. , Raccosta, S. , Romancino, D. P. , Noto, R. , Touzet, N. , Manno, M. , Di Schiavi, E. , Bongiovanni, A. , & Adamo, G. (2022). Extracellular vesicles from microalgae: Uptake studies in human cells and *Caenorhabditis elegans* . Frontiers in Bioengineering and Biotechnology, 10, 830189. 10.3389/fbioe.2022.830189 35402397 PMC8987914

[jex270025-bib-0051] Pirolli, N. H. , Bentley, W. E. , & Jay, S. M. (2021). Bacterial extracellular vesicles and the gut‐microbiota brain axis: Emerging roles in communication and potential as therapeutics. Advanced Biology, 5, e2000540. 33857347 10.1002/adbi.202000540

[jex270025-bib-0052] Pretre, G. , Lapponi, M. J. , Atzingen, M. V. , Schattner, M. , Nascimento, A. L. , & Gómez, R. M. (2013). Characterization of LIC11207, a novel leptospiral protein that is recognized by human convalescent sera and prevents apoptosis of polymorphonuclear leukocytes. Microbial Pathogenesis, 56, 21–28. 10.1016/j.micpath.2012.10.002 23092690

[jex270025-bib-0053] Préville, X. , Ladant, D. , Timmerman, B. , & Leclerc, C. (2005). Eradication of established tumors by vaccination with recombinant Bordetella pertussis adenylate cyclase carrying the human papillomavirus 16 E7 oncoprotein. Cancer Research, 65, 641–649. 15695409

[jex270025-bib-0054] Ragaza, J. A. , Hossain, M. S. , Meiler, K. A. , Velasquez, S. F. , & Kumar, V. (2020). A review on Spirulina: Alternative media for cultivation and nutritive value as an aquafeed. Reviews in Aquaculture, 12, 2371–2395. 10.1111/raq.12439

[jex270025-bib-0055] Reimer, S. L. , Beniac, D. R. , Hiebert, S. L. , Booth, T. F. , Chong, P. M. , Westmacott, G. R. , Zhanel, G. G. , & Bay, D. C. (2021). Comparative analysis of outer membrane vesicle isolation methods with an *Escherichia coli tolA* mutant reveals a hypervesiculating phenotype with outer‐inner membrane vesicle content. Frontiers in Microbiology, 12, 628801. 10.3389/fmicb.2021.628801 33746922 PMC7973035

[jex270025-bib-0056] Ristow, L. C. , & Welch, R. (2019). A. RTX toxins ambush immunity's first cellular responders. Toxins, 11, 720. 31835552 10.3390/toxins11120720 PMC6950748

[jex270025-bib-0057] Ryan, A. , Lynch, M. , Smith, S. M. , Amu, S. , Nel, H. J. , McCoy, C. E. , Dowling, J. K. , Draper, E. , O'Reilly, V. , McCarthy, C. , O'Brien, J. , Ní Eidhin, D. , O'Connell, M. J. , Keogh, B. , Morton, C. O. , Rogers, T. R. , Fallon, P. G. , O'Neill, L. A. , Kelleher, D. , & Loscher, C. E. (2011). A role for TLR4 in Clostridium difficile infection and the recognition of surface layer proteins. PLoS Pathogens, 7(6), e1002076. 10.1371/journal.ppat.1002076 21738466 PMC3128122

[jex270025-bib-0058] Rybak, K. , & Robatzek, S. (2019). Functions of extracellular vesicles in immunity and virulence. Plant Physiology, 179, 1236–1247. 30705070 10.1104/pp.18.01557 PMC6446742

[jex270025-bib-0059] Sanchez, Y. , Ionescu‐Matiu, I. , Dreesman, G. R. , Kramp, W. , Six, H. R. , Hollinger, F. B. , & Melnick, J. L. (1980). Humoral and cellular immunity to hepatitis B virus‐derived antigens: Comparative activity of Freund complete adjuvant alum, and liposomes. Infection and Immunity, 30(3), 728–733. 10.1128/iai.30.3.728-733.1980 7014445 PMC551376

[jex270025-bib-0060] Satchell, K. J. (2011). Structure and function of MARTX toxins and other large repetitive RTX proteins. Annual Review of Microbiology, 65, 71–90. 10.1146/annurev-micro-090110-102943 21639783

[jex270025-bib-0061] Sayers, E. W. , Bolton, E. E. , Brister, J. R. , Canese, K. , Chan, J. , Comeau, D. C. , Farrell, C. M. , Feldgarden, M. , Fine, A. M. , Funk, K. , Hatcher, E. , Kannan, S. , Kelly, C. , Kim, S. , Klimke, W. , Landrum, M. J. , Lathrop, S. , Lu, Z. , Madden, T. L. , … Sherry, S. T. (2023). Database resources of the National Center for Biotechnology Information in 2023. Nucleic Acids Research, 51(D1), D29–D38. 10.1093/nar/gkac1032 36370100 PMC9825438

[jex270025-bib-0062] Schätzle, H. , Brouwer, E. M. , Liebhart, E. , Stevanovic, M. , & Schleiff, E. (2021). Comparative Phenotypic analysis of anabaena sp. PCC 7120 mutants of porinlike genes. Journal of Microbiology and Biotechnology, 31, 645–658. 33879642 10.4014/jmb.2103.03009 PMC9705863

[jex270025-bib-0063] Schorey, J. S. , Cheng, Y. , Singh, P. P. , & Smith, V. L. (2015). Exosomes and other extracellular vesicles in host–pathogen interactions. EMBO Reports, 16, 24–43. 25488940 10.15252/embr.201439363 PMC4304727

[jex270025-bib-0064] Selmi, C. , Leung, P. S. , Fischer, L. , German, B. , Yang, C. Y. , Kenny, T. P. , Cysewski, G. R. , & Gershwin, M. E. (2011). The effects of Spirulina on anemia and immune function in senior citizens. Cellular & Molecular Immunology, 8(3), 248–254. 10.1038/cmi.2010.76 21278762 PMC4012879

[jex270025-bib-0065] Sikder, S. , Rush, C. M. , Govan, B. L. , Alim, M. A. , & Ketheesan, N. (2020). Anti‐streptococcal antibody and T‐cell interactions with vascular endothelial cells initiate the development of rheumatic carditis. Journal of Leukocyte Biology, 107, 263–271. 31617241 10.1002/JLB.4MA0919-096RR

[jex270025-bib-0066] Sili, C. , Torzillo, G. , & Vonshak, A. (2012). Arthrospira (Spirulina). Ecology of Cyanobacteria II: Their Diversity in Space and Time, 9789400738553, 677–705.

[jex270025-bib-0067] Silva, M. , Videira, P. A. , & Sackstein, R. (2018). E‐selectin ligands in the human mononuclear phagocyte system: Implications for infection, inflammation, and immunotherapy. Frontiers in immunology, 8, 1878. 29403469 10.3389/fimmu.2017.01878 PMC5780348

[jex270025-bib-0068] Silva, M. R. O. B. D. , M da Silva, G. , Silva, A. L. F. D. , Lima, L. R. A. , Bezerra, R. P. , & Marques, D. A. V. (2021). Bioactive compounds of *Arthrospira* spp. (Spirulina) with potential anticancer activities: A systematic review. ACS Chemical Biology, 16(11), 2057–2067. 10.1021/acschembio.1c00568 34597512

[jex270025-bib-0069] Singh, V. , Kaur, R. , Kumari, P. , Pasricha, C. , & Singh, R. (2023). ICAM‐1 and VCAM‐1: Gatekeepers in various inflammatory and cardiovascular disorders. Clinica Chimica Acta, 548, 117487. 10.1016/j.cca.2023.117487 37442359

[jex270025-bib-0070] Stanly, C. , Fiume, I. , Capasso, G. , & Pocsfalvi, G. (2016). Isolation of exosome‐like vesicles from plants by ultracentrifugation on sucrose/deuterium oxide (D2O) density cushions. Methods in Molecular Biology, 1459, 259–269. 10.1007/978-1-4939-3804-9_18 27665565

[jex270025-bib-0071] Stecher, G. , Tamura, K. , & Kumar, S. (2020). Molecular evolutionary genetics analysis (MEGA) for macOS. Molecular Biology and Evolution, 37, 1237–1239. 31904846 10.1093/molbev/msz312 PMC7086165

[jex270025-bib-0072] Supek, F. , Bošnjak, M. , Škunca, N. , & Šmuc, T. (2011). REVIGO summarizes and visualizes long lists of gene ontology terms. PLoS ONE, 6, e21800. 21789182 10.1371/journal.pone.0021800 PMC3138752

[jex270025-bib-0073] Tamura, K. , & Nei, M. (1993). Estimation of the number of nucleotide substitutions in the control region of mitochondrial DNA in humans and chimpanzees. Molecular Biology and Evolution, 10(3), 512–526. 10.1093/oxfordjournals.molbev.a040023 8336541

[jex270025-bib-0074] Toyofuku, M. , Nomura, N. , & Eberl, L. (2018). Types and origins of bacterial membrane vesicles. Nature Reviews Microbiology, 17(1), 13–24. 10.1038/s41579-018-0112-2 30397270

[jex270025-bib-0075] Usui, K. , Yamamoto, H. , Oi, T. , Taniguchi, M. , Mori, H. , & Fujita, Y. (2022). Extracellular vesicle‐mediated secretion of protochlorophyllide in the cyanobacterium *Leptolyngbya boryana* . Plants, 11(7), 910. 10.3390/plants11070910 35406890 PMC9003413

[jex270025-bib-0076] van den Berg, B. (2012). Structural basis for outer membrane sugar uptake in pseudomonads. Journal of Biological Chemistry, 287, 41044–41052. 23066028 10.1074/jbc.M112.408518 PMC3510807

[jex270025-bib-0077] van Niel, G. , D'Angelo, G. , & Raposo, G. (2018). Shedding light on the cell biology of extracellular vesicles. Nature Reviews Molecular Cell Biology, 19, 213–228. 29339798 10.1038/nrm.2017.125

[jex270025-bib-0078] Vieira, M. L. , D'Atri, L. P. , Schattner, M. , Habarta, A. M. , Barbosa, A. S. , de Morais, Z. M. , Vasconcellos, S. A. , Abreu, P. A. , Gómez, R. M. , & Nascimento, A. L. (2007). A novel leptospiral protein increases ICAM‐1 and E‐selectin expression in human umbilical vein endothelial cells. FEMS Microbiology Letters, 276(2), 172–180. 10.1111/j.1574-6968.2007.00924.x 17956423

[jex270025-bib-0079] Wiklander, O. P. B. , Brennan, M. Á. , Lötvall, J. , Breakefield, X. O. , & El Andaloussi, S. (2019). Advances in therapeutic applications of extracellular vesicles. Science Translational Medicine, 11(492), eaav8521. 10.1126/scitranslmed.aav8521 31092696 PMC7104415

[jex270025-bib-0080] Wu, Q. , Liu, L. , Miron, A. , Klímová, B. , Wan, D. , & Kuča, K. (2016). The antioxidant, immunomodulatory, and anti‐inflammatory activities of Spirulina: An overview. Archives of Toxicology, 90(8), 1817–1840. 10.1007/s00204-016-1744-5 27259333

[jex270025-bib-0081] Wylie, J. L. , & Worobec, E. A. (1995). The OprB porin plays a central role in carbohydrate uptake in Pseudomonas aeruginosa. Journal of Bacteriology, 177, 3021–3026. 7768797 10.1128/jb.177.11.3021-3026.1995 PMC176988

[jex270025-bib-0082] Yang, J. , Li, B. , Yang, D. , Wu, J. , Yang, A. , Wang, W. , Lin, F. , Wan, X. , Li, Y. , Chen, Z. , Lv, S. , Pang, D. , Liao, W. , Meng, S. , Lu, J. , Guo, J. , Wang, Z. , & Shen, S. (2023). The immunogenicity of Alum+CpG adjuvant SARS‐CoV‐2 inactivated vaccine in mice. Vaccine, 41(41), 6064–6071. 10.1016/j.vaccine.2023.08.061 37640568

[jex270025-bib-0083] Yin, H. , Chen, C. Y. , Liu, Y. W. , Tan, Y. J. , Deng, Z. L. , Yang, F. , Huang, F. Y. , Wen, C. , Rao, S. S. , Luo, M. J. , Hu, X. K. , Liu, Z. Z. , Wang, Z. X. , Cao, J. , Liu, H. M. , Liu, J. H. , Yue, T. , Tang, S. Y. , & Xie, H. (2019). *Synechococcus elongatus* PCC7942 secretes extracellular vesicles to accelerate cutaneous wound healing by promoting angiogenesis. Theranostics, 9(9), 2678–2693. 10.7150/thno.31884 31131061 PMC6525994

[jex270025-bib-0084] Zhao, J. , Bi, W. , Xiao, S. , Lan, X. , Cheng, X. , Zhang, J. , Lu, D. , Wei, W. , Wang, Y. , Li, H. , Fu, Y. , & Zhu, L. (2019). Neuroinflammation induced by lipopolysaccharide causes cognitive impairment in mice. Scientific Reports, 9(1), 5790. 10.1038/s41598-019-42286-8 30962497 PMC6453933

[jex270025-bib-0085] Zhao, T. , Cai, Y. , Jiang, Y. , He, X. , Wei, Y. , Yu, Y. , & Tian, X. (2023). Vaccine adjuvants: Mechanisms and platforms. Signal Transduction and Targeted Therapy, 8(1), 283. 10.1038/s41392-023-01557-7 37468460 PMC10356842

