# Supplementary material for: Characterization of Spirulina‐derived extracellular vesicles and their potential as a vaccine adjuvant
Source: J Extracell Biol. 2024 Dec 12;3(12):e70025. doi: 10.1002/jex2.70025 (PMC11635480; doi:10.1002/jex2.70025)
Supplement: Supplementary file 1 — Supporting Information [file JEX2-3-e70025-s001.docx]

**Supplementary Documents**

S-Fig. 1, 2, and 3; and S-Table 1 are presented in this file.
S-Tables 2, 3, and 4 are in Excel file formats that are uploaded separately.


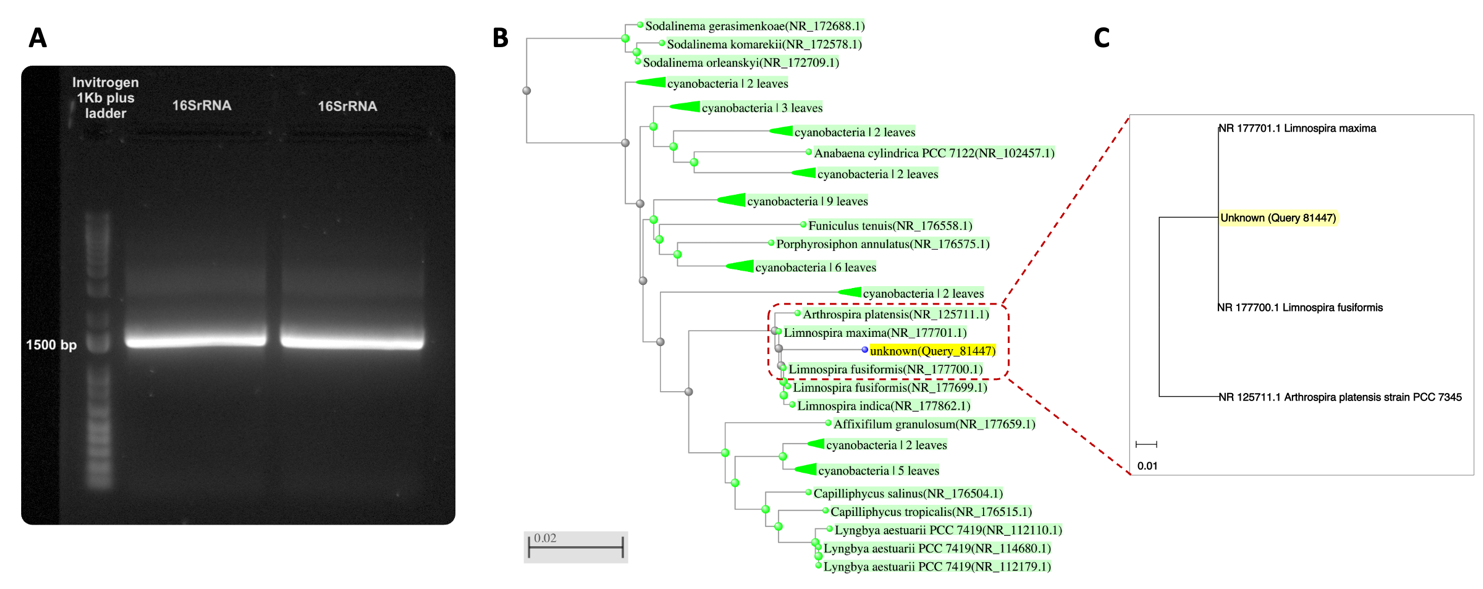


**Supplementary Figure. 1:** The phylogenetic analysis of the Spirulina subject showed that it was closely related to *Limnospira* *maxima* and *fusiformis* spp. A. PCR result for amplification of partial 16SrRNA sequence. B. Neighbour-Joining Tree comparing the query sample (highlighted) with other cyanobacteria. C. Maximum Likelihood tree, comparing the query sample with three closely related species.


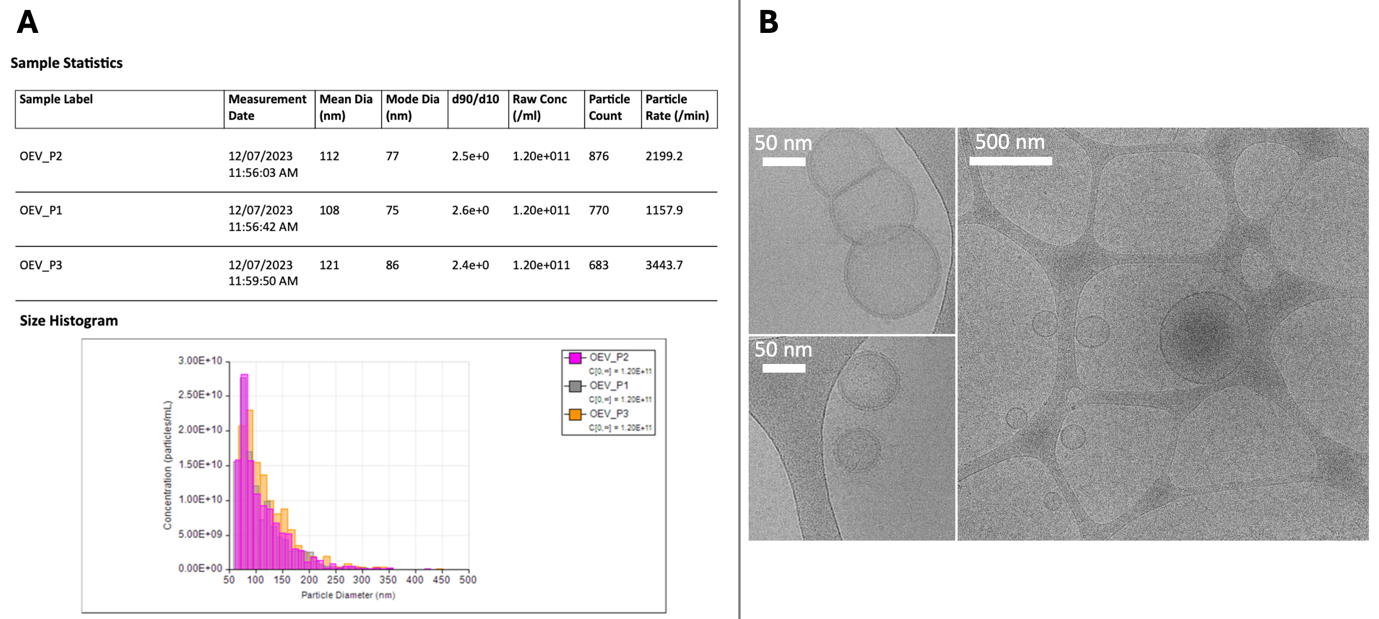


Supplementary Figure 2: Orange EV characterization. A. Size distribution and concentration of orange EVs measured by a tuneable resistive pulse sensing technology using Exoid (Izon). B. Cryo-transmission electron micrographs of orange EVs.


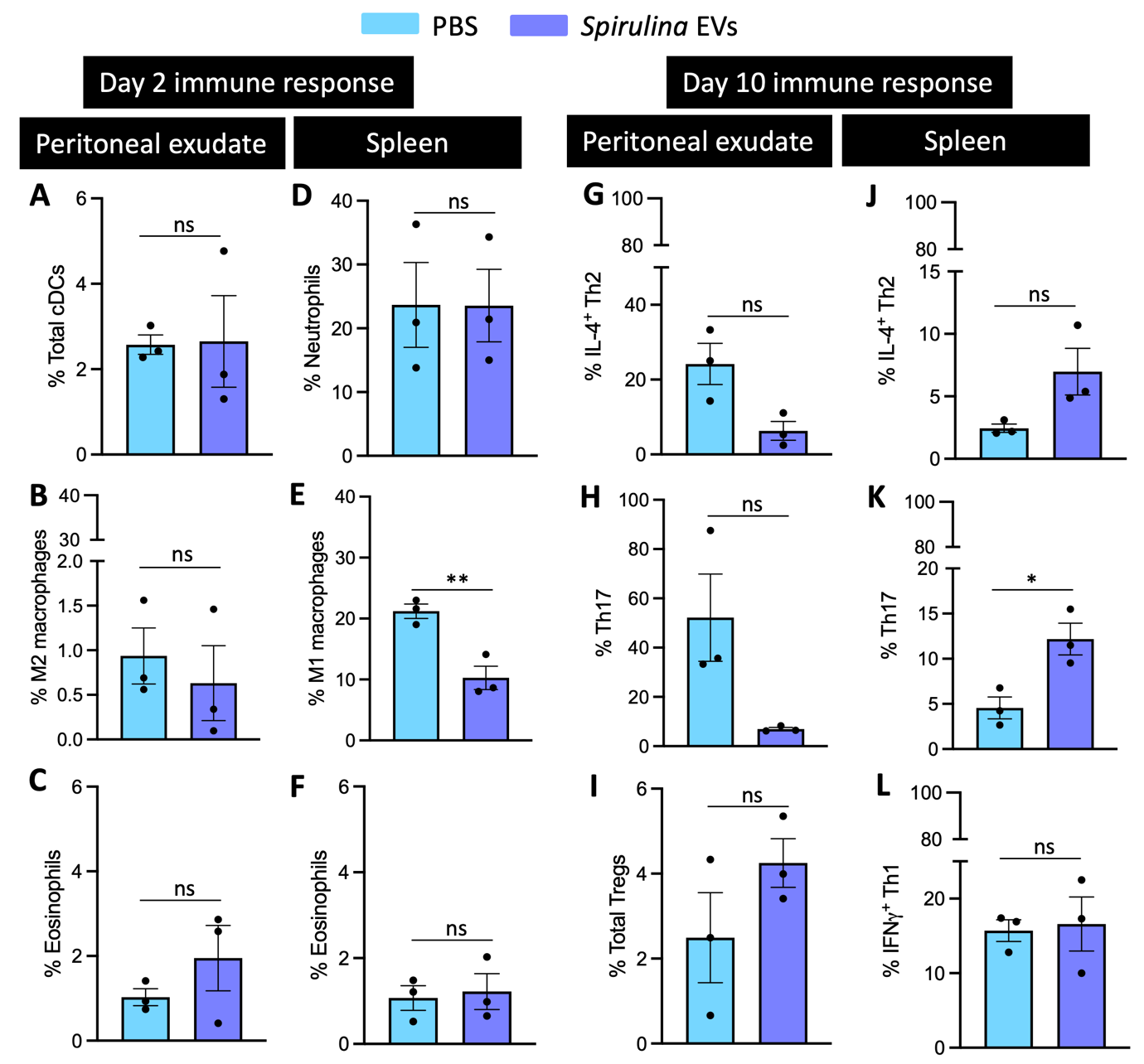


**Supplementary Figure 3:** Innate and adaptive immune response to Spirulina EVs. Day 2 early total cDC (Live CD11c^+^) (A), M2 macrophage (Live CD11b^+^ F4/80^+^ CD206^+^) (B) and eosinophil (Live CD11b^+^ SiglecF^+^) response (C) to SPEVs at the site of injection, and neutrophil (Live CD11b^+^ Ly6G^+^) (D), M1 macrophage (Live CD11b^+^ F4/80^+^ CD11c^+^) (E), and eosinophil response (F) in spleen. Day 10 Th cell response represents IL4^+^ Th2 cell (of Live CD3^+^ CD4^+^ CD44^+^) (G), IL-17^+^ Th17 cell (of Live CD3^+^ CD4^+^ CD44^+^) (H), and total Tregs (Live CD3^+^ CD4^+^ CD25^+^ FoxP3^+^) (I) in peritoneal exudate, and IL-4^+^ Th2 cell (J), IL-17^+^ Th17 cell (K), and IFNγ^+^ Th1 cell (of Live CD3^+^ CD4^+^ CD44^+^) response (L) in spleen. Error bars represent standard errors of the mean. Statistical analysis by t-test, p* <0.05, ** <0.01, ns: not significant.


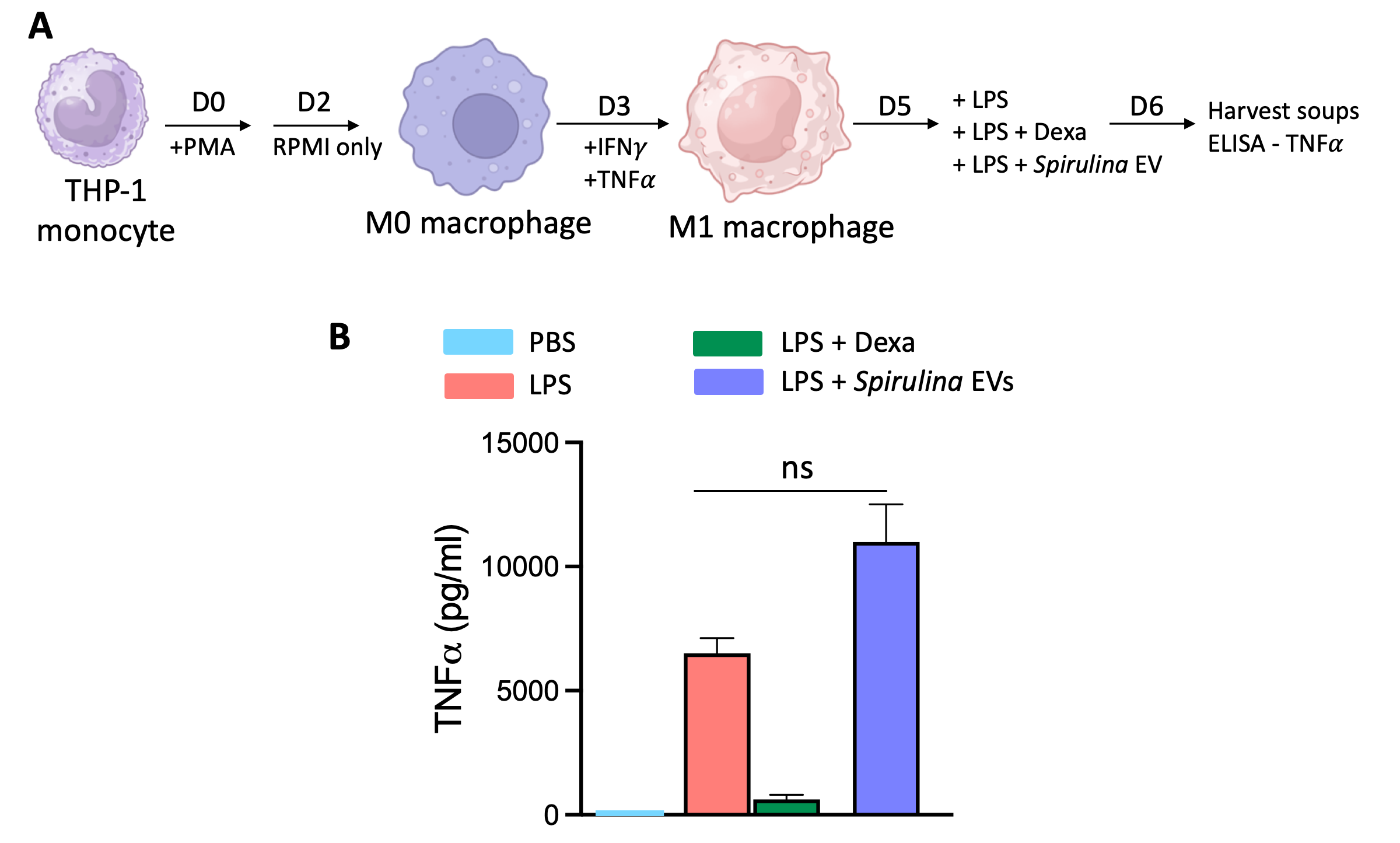


**Supplementary Figure 4:** Assessment of TNF𝛼 secretion by THP-1 derived macrophages stimulated with SPEVs. A) THP-1 monocytes were stimulated with PMA followed by PMA starvation and IFN𝛾 and TNF𝛼 stimulation to polarise to M1 macrophages. Polarised macrophages were incubated with LPS ± *Spirulina* EVs. Stimulation with LPS + Dexamethasone was used as positive control. B) Cell culture supernatants were tested for TNF𝛼 by ELISA. *Spirulina* EVs insignificantly increased TNF𝛼 secretion. Error bars represent standard errors of the mean. Statistical analysis by t-test, ns: not significant.

**Supplementary Table 1**. The nucleotide pairwise distance for the three closely related species to the Spirulina sample (Unknown), calculated by MEGA 11 software.

|  | NR_177700.1  *Limnospira fusiformis* | NR_177701.1  *Limnospira maxima* | NR_125711.1  *Arthrospira platensis* | Unknown  (Query_81447) |
| --- | --- | --- | --- | --- |
| NR_177700.1  *Limnospira fusiformis* |  |  |  |  |
| NR_177701.1  *Limnospira maxima* | 0.0007741157 |  |  |  |
| NR_125711.1  *Arthrospira platensis* | 0.0666974263 | 0.0039105253 |  |  |
| Unknown (Query 81447) | 0.0000000000 | 0.0000000000 | 0.0143512949 |  |
